# Targeting ferroptosis in cancer: from mechanistic insights to therapeutic approaches

**DOI:** 10.1186/s43556-026-00416-5

**Published:** 2026-03-03

**Authors:** Junqi Wang, Dawei Guo, Shanxiang Jiang, Wenda Wu, Xiuge Gao

**Affiliations:** 1 MOE Joint International Research Laboratory of Animal Health and Food Safety, College of Veterinary Medicine, Nanjing Agricultural University, Nanjing, 210095 China; 2 Engineering Center of Innovative Veterinary Drugs, Center for Veterinary Drug Research and Evaluation, College of Veterinary Medicine, Nanjing Agricultural University, 1 Weigang, Nanjing, 210095 China; 3 School of Food and Biological Engineering, Hefei University of Technology, Hefei, 230009 China; 4 Department of Chemistry, Faculty of Science, University of Hradec Kralove, 50003 Hradec Kralove, Czech Republic

**Keywords:** Ferroptosis, Cancer, Lipid peroxidation, Iron, Tumor microenvironment, Immunotherapy

## Abstract

Ferroptosis is a promising programmed cell death modality for cancer therapy, driven by iron overload and the accumulation of phospholipid peroxides that culminate in lethal membrane damage. Over the past decade, emerging evidence supports the concept that ferroptosis can be harnessed as an effective strategy to suppress tumor growth, particularly in therapy-resistant cancer cells undergoing epithelial–mesenchymal transition and in cancer stem cells. Given that ferroptosis is mechanistically and morphologically different from other known programmed cell death forms, increasing critical findings have shed light on mechanisms by which ferroptosis is regulated, and context-dependent cancer phenotype which is clinical relevant to ferroptosis. In this review, we summarize the basic biology of ferroptosis, including iron regulation and lipid metabolism, as well as key molecular mechanisms such as the system Xc⁻-GSH-GPX4, NADPH-FSP1-CoQ10 and GCH1-BH4 axis in fighting cancer. We also discuss crosstalk between ferroptosis and cuproptosis, disulfidptosis and autophagy, and outline how ferroptosis shapes the tumor immune microenvironment and responses to immunotherapy. More importantly, we highlight the clinical potential of ferroptosis induction via chemotherapy, radiotherapy, immunotherapy and nanomedicine-based delivery strategies, while summarizing common resistance mechanisms and safety considerations. Finally, we outline major challenges and pressing questions for clinical translation, including what are the molecular bases of ferroptosis, how can ferroptosis be leveraged for cancer therapy, how can ferroptosis be integrated with conventional therapies, and how to balance benefits and risks of ferroptosis-based therapy. Collectively, this review connects mechanistic insights with actionable intervention points for developing ferroptosis-based cancer therapies.

## Introduction

Recent decades have witnessed breakthroughs in cancer therapy, including the development of targeted therapy, immunotherapy, cell therapy, and nanomedicine. Although these innovative solutions show promising approaches in cancer treatment, chemotherapy is still the commonly used clinical treatment. However, tumor cells often develop resistance to conventional therapies, limiting their efficacy and leading to treatment failure and cancer-related mortality [1]. Because mainstream tumor-suppressing chemotherapies heavily depend on apoptosis to kill tumor cells or inhibit tumor growth [2], the discovery of novel, nonapoptotic cell death modalities to suppress malignant tumor growth is clinically important. Recently, a novel nonapoptotic form of cell death, ferroptosis, has been identified and described as a previously unknown regulatory mechanism that suppresses tumor growth via iron-dependent lipid peroxidation [3]. The term ferroptosis was first coined by Brent Stockwell and colleagues in 2012 on the basis of a high-throughput screening of novel cancer therapeutic compounds used to treat engineered human cancer cells [3]. The morphological, biochemical, and genetic features of ferroptosis make it different from previously established regulated cell death (RCD) modalities such as apoptosis, necrosis and autophagic cell death [3]. Two hallmarks of ferroptosis are excessive intracellular accumulation of iron and lipid peroxidation in the course of the cell death process. In addition, multiple metabolic pathways related to iron, lipids, oxidoreductases and amino acids determine the sensitivity of cells to ferroptosis [4].

Since its initial description, ferroptosis has been implicated in diverse diseases, including neurodegenerative disorders, ischemic organ injury, and therapy-resistant cancers [5]. Over the past few years, key molecules, regulatory mechanisms, chemical inducers and inhibitors of ferroptosis have been identified, and therapeutic technologies targeting excessively active iron-dependent lipid peroxidation pathways have been developed [5]. Intriguingly, multiple cancer-associated genes and signaling pathways regulate ferroptosis, and many therapy-resistant cancer cells show heightened sensitivity to its induction [6, 7]. Notably, primary cancers and metastatic cancers have both been shown to be sensitive to ferroptosis induction due to their high demand for iron and unique metabolism states, which differ from those of normal cells [7]. Interestingly, cancer stem cells (CSCs) have been found to be susceptible to ferroptosis inducers (FINs), suggesting that ferroptosis plays a potential important role in eradicating and overcoming cancer resistance to normal therapeutics by inducing CSC death [8]. In recent years, several inspiring findings revealed that the radiotherapy-induced ferroptosis of cancer cells correlated with longer patient survival [9]. Additionally, ferroptosis has been closely associated with cancer immunotherapy [10]. Radiotherapy combined with immunotherapy promotes cancer cell lipid peroxidation and ferroptosis, enabling further development of an effective combined targeted cancer therapy strategy for overcoming resistant cancers [11]. Recent and rapidly growing investigations into ferroptosis mechanisms and related therapeutics have expanded the avenues for developing ferroptosis as a novel treatment against cancer.

Here, we review the molecular mechanisms and context-dependent regulation of ferroptosis in cancer and summarize emerging therapeutic strategies to exploit this vulnerability. We also discuss key translational challenges and outline priorities for advancing ferroptosis-based interventions toward clinical cancer treatment.

## Mechanistic insights into ferroptosis regulation

### Iron metabolism and ferroptosis

As the name implies, ferroptosis is an iron-dependent process. However, the key role played by iron and the regulation of iron metabolism have only recently been described, especially in the context of cancer suppression [12]. Unlike normal cells, malignant cells exhibit a heightened dependence on iron, which supports their rapid proliferation and metastatic potential. The dependence of cancer cells on iron has been dubbed “iron addiction” [13]. This increased iron demand partly underlies the sensitivity of cancer cells to ferroptosis, suggesting that iron metabolism may be closely linked to ferroptosis initiation in cancer therapy. In addition, iron overload has been shown to induce ferroptosis due to the high levels of iron-dependent lipid peroxides in numerous types of malignant cells, including liver cancer, clear kidney cancer and certain other cancer cells that have acquired drug resistance [8]. Moreover, the levels of iron that is imported, stored, degraded and exported determine cancer cell sensitivity to ferroptosis. Extracellular ferric ions are imported into cells by transferrin and transferrin receptor 1 (TFRC), and the internalized iron-carrying complex dissociates in acidic organelles such as lysosomes and late endosomes, releasing free ferric irons [14]. Intriguingly, CD44, a transmembrane glycoprotein, mediates iron-bound hyaluronates endocytosis in the mesenchymal state of cancer cells, performing as a potential predictor of cancer cells vulnerable to ferroptosis [15]. The newly generated ferric irons are reduced via metalloreductase six transmembrane epithelial antigen of prostate 3 (STEAP3) and transferred to the cytoplasm by divalent metal transporter 1 (DMT1) or solute carrier family 11, member 2 (SLC11A2). These free irons form a labile iron pool (LIP), which initiates the Fenton reaction phase of a chain reaction that leads to the generation of lipid peroxide that targets membranes. Many cellular processes can alter the sensitivity of cancer cells to ferroptosis by regulating the contents of the labile iron pool [12]. For example, iron-regulatory protein 1 and 2 (IRP1 and IRP2) coordinate to regulate iron uptake, transport, storage and turnover by binding to several iron-responsive genes. Recently, the Possemato group showed that abrogation of iron-sulfur cluster synthesis activated IRP2 and promoted the ferroptosis sensitivity of MDA-MB-231 cells, which was mediated through IRP1- and FBXL5-independent pathways [16]. Furthermore, the upregulation of TFRC expression and downregulation of ferritin heavy chain 1 (FTH1) and ferritin light chain (FTL) expression contributed to the elevation of iron content, resulting in the susceptibility of melanoma cells to ferroptosis. In contrast, the increased expression of CDGSH iron sulfur domain 1 (CISD1), CISD3 and the cysteine desulfurase nitrogen fixation 1 (NFS1), which are involved in iron-sulfur cluster biogenesis, led to the ferroptotic insensitivity of hepatocellular carcinoma (HCC), HT1080 cells and non-small-cell lung cancer (NSCLC) cells [17, 18]. Hypoxia-inducible factor-2α (HIF-2α) activation increased the cellular iron level and potentiated oxidative stress-related death in colorectal cancer [19]. In addition to these above iron regulators, mediator of ERBB2-driven cell motility 1 (MEMO1) has been demonstrated as an iron-binding protein, which modulates iron homeostasis in cancer cells and promotes ferroptosis via enhancing labile iron pool [20].

Notably, the abundance of intracellular ferritin governs the sensitivity of cancer cells to ferroptosis, and a higher ferritin level indicates more stored ferric ions and greater cellular resistance to ferroptotic death [21, 22]. Iron indirectly produces lipid peroxidation by catalyzing iron-containing enzymes, and lipoxygenases (LOXs) play significant roles during ferroptosis. Furthermore, heme degradation induced by heme oxygenase-1 (HO-1)-elevated iron levels promoted ferroptosis [23], although another study demonstrated conflicting data, showing that HO-1 suppressed the ferroptosis of HCC cells [24]. Kinases also play critical roles in cancer cell ferroptosis. For instance, Chi et al. demonstrated a novel regulatory mechanism through which the serine/threonine kinase ATM downregulated the expression of FTH1, FTL and FPN1, and inhibited the nuclear translocation of metal-regulatory transcription factor 1 (MTF1), resulting in the elevation of the labile iron pool and promotion of MDA-MB-231 breast cancer cell ferroptosis [25]. Additionally, aberrant iron metabolism has been shown to be a prominent feature of CSCs, suggesting the potential development of alternative methods to eliminate CSCs via the induction of ferroptosis.

In addition to iron uptake and storage, the regulation of iron export is a critical determinant of ferroptosis sensitivity. Hepcidin, a liver-derived peptide hormone that is also produced by tumor cells and stromal cells in several cancers, acts as the systemic “gatekeeper” of iron homeostasis by binding to the iron exporter ferroportin (FPN1) and triggering its internalisation and lysosomal degradation. Downregulation of FPN1 decreases cellular iron efflux, increases intracellular labile iron levels and thereby favours iron-catalysed lipid peroxidation and ferroptosis [26]. In many malignancies, including breast, colorectal and hepatocellular carcinoma, tumor- or stromal-derived hepcidin contributes to cancer progression by retaining iron within tumor cells and tumor-associated macrophages, while loss of FPN1 expression correlates with aggressive behaviour and poor prognosis [27]. Hepcidin–ferroportin signalling also intersects with the intracellular iron regulatory protein/iron-responsive element (IRP/IRE) system: hepcidin-mediated FPN1 degradation elevates the labile iron pool and inactivates IRP1/2, which in turn relieves translational repression of ferritin and FPN1 while reducing transferrin receptor synthesis, forming a feedback loop that buffers extreme iron fluctuations [28]. In cancer cells, dysregulation of this hepcidin–FPN1–IRP/IRE axis can therefore either enhance or restrain ferroptosis, depending on whether iron retention is coupled to sufficient lipid peroxidation, adding another layer of complexity to iron metabolism-driven ferroptotic responses.

Conversely, some mechanisms enhance iron export from cells, thereby increasing tumor cell resistance to ferroptosis. Brown et al. recently revealed that prominin 2 promoted the formation of multivesicular bodies and exosomes containing ferritin, which transported iron out of cells, driving ferroptosis resistance [29]. Furthermore, ferroportin led to intracellular iron export, which downregulated the ferroptosis of neuroblastoma SH-SY5Y cells, indicating that ferroportin inhibition may lead to novel chemosensitization in neuroblastoma [30]. Mechanistically, the ubiquitin (Ub)-specific protease 35 (USP35) stabilizes ferroportin, thereby promoting iron accumulation and sensitizing human lung cancer cells to ferroptosis [31]. Similarly, heat shock protein β−1 (HSPβ−1) prevents cancer cells from ferroptosis by decreasing intracellular iron levels as well as inhibiting lethal lipid peroxidation [32]. In addition, the critical transcription factor NRF2 regulating redox homeostasis has been recently confirmed to maintain iron homeostasis and to confer ovarian cancer cells resistant to ferroptosis in which NRF2 controls ferritin synthesis and degradation through HERC2 (E3 ubiquitin ligase for NCOA4 and FBXL5), VAMP8 (vesicle associated membrane protein 8) [33].

Signaling molecules that regulate intracellular iron levels influence ferroptosis susceptibility by modulating iron homeostasis. For example, the Ser/Thr protein kinase GSK-3β positively modulated ferroptosis by increasing labile iron levels in HeLa cells [34]. Similarly, glutaredoxin 5 (GLRX5) inhibition predisposed therapy-resistant HNC cells to ferroptosis by activating the iron-starvation response and boosting intracellular free iron levels [35]. A regulator named coatomer protein complex subunit zeta 1 (COPZ1) has been demonstrated to be a therapeutic target candidate because knocking down its expression increased iron levels via NCOA4-/FTH1-induced ferritinophagy in glioblastoma (GBM) cells [36]. MYCN expression sensitizes neuroblastoma cells to ferroptosis inducers by reprogramming iron metabolism and upregulating transferrin receptor (TFRC) activity, thereby increasing labile iron [37]. Additionally, the Ub proteasome system (UPS) plays a critical role in regulating ferroptosis by controlling intracellular labile iron levels, and tribbles homolog 2 (TRIB2) has been found to desensitize liver cancer cells to ferroptosis through beta-transducin repeat containing protein (βTrCP) E3 ubiqutin protein ligase -mediated TFRC ubiquitination [38]. In addition to the effects of iron-related molecular signaling, regulators of Ca^2+^ levels play critical roles in ferroptosis. A recent study demonstrated that MS4A15, a calcium regulator on the ER, conferred ferroptosis resistance on multiple types of cancer cells by depleting luminal Ca^2+^ stores and reprogramming membrane PLs [39]. Similarly, another recent study found that the regulation of zinc abundance determined the ferroptosis rate of breast and renal cancer cells; specifically, the zinc transporter ZIP7 promoted ferroptosis by inducing ER stress and dysregulating the expression of HERPUD1 and ATF3 [40]. Moreover, certain molecular pathways support tumorigenesis by constitutively suppressing ferroptosis, as illustrated in liver cancer by the LIFR-NFκB-LCN2 axis [41].

**Fig. 1:**
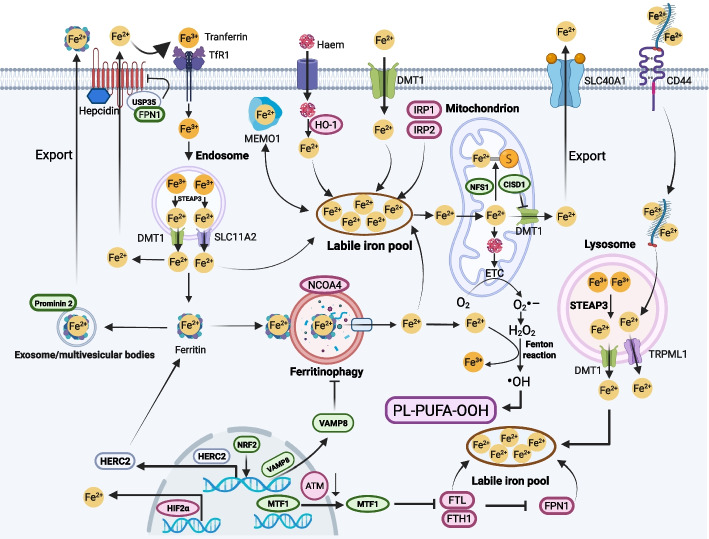
Iron overload sensitizes cancer cells to ferroptosis. Ferroptosis induction depends on the intracellular accumulation of free iron (the labile iron pool), and the processes involved in the generation and utilization of irons determine the sensitivity of cancer cells to ferroptosis. Extracellular ferric ions bind and are transported by transferrin (TF) and are recognized by transferrin receptor 1 (TFRC). After endocytosis in endosomes and reduction by STEAP3, Fe^2+^ is released into the cytosol, contributing to the labile iron pool. Free iron is transported from the extracellular medium to the cytosol by divalent metal transporter 1 (DMT1) and is directly added to the labile iron pool. Iron can also be exported from mitochondria and lysosomes into the cytosol. Ferritinophagy is a mechanism leading to Fe^2+^ release via autophagy-dependent ferritin degradation. This free iron is necessary for the Fenton reaction, which promotes lipid peroxidation, leading to lethal ferroptosis. In contrast, iron export from the tumor cell cytosol prevents iron overloading and inhibits ferroptosis. For example, ferritin captured in multivesicular bodies or exosomes can be transferred extracellularly, leading to cellular resistance to ferroptosis. Importantly, several genes, including NRF2, HIF2α and MTF1, are involved in the regulation of intracellular free irons and influence the vulnerability of cancer cells to ferroptosis

Overall, iron metabolism is tightly regulated to maintain homeostasis, and the labile iron pool is a key determinant of ferroptosis sensitivity (Fig. 1). Accordingly, strategies that increase intracellular labile iron in malignant cells may potentiate ferroptosis for cancer therapy.

### Lipid metabolism and ferroptosis

Lipid peroxidation is a hallmark of ferroptosis and drives ferroptotic cell death through membrane damage [42]. During lipid metabolism, the bis-allylic hydrogen atoms of polyunsaturated fatty acids (PUFAs) render them susceptible to peroxidation, a process essential for ferroptosis [42]. Thus, the abundance and localization of PUFAs determine the degree of lipid peroxidation as well as the sensitivity of cells to ferroptosis. Only specific types of phospholipids (PLs) peroxides trigger ferroptosis, and these free PUFAs must be esterified into membrane PLs to generate lipid signals [43]. Lipidomic studies indicate that phosphatidylethanolamines containing arachidonic acid (AA) or adrenic acid (AdA) are key phospholipids that undergo peroxidation during ferroptosis [43]. Therefore, membranes containing a high level of PUFA-containing PLs (PUFA-PLs) are particularly vulnerable to peroxidation. Intriguingly, diacyl-PUFA phosphatidylcholines (PC-PUFA_2_S) is a newly identified driver of lipid peroxidation and ferroptosis by inducing mitochondrial ROS, indicating the diversity of phospholipid species which are critical for ferroptotic death [44]. Although lipid peroxidation can occur on various membranes, recent evidence indicates that the endoplasmic reticulum (ER) membrane is a primary site of peroxidation during ferroptosis, highlighting the ER’s key role in initiating this cell death [45]. Elevated levels of free fatty acids and a high fatty acid oxidation rate are related to the regulation of progesterone receptor membrane component 1 (PGRMC1), which renders paclitaxel-tolerant persister cancer cells (PCC) sensitive to ferroptosis via lipophagy and tubulin detyrosination [46]. Certain cancer cells exhibit enhanced sensitivity to ferroptosis [6, 47]. Notably, in the acidic tumor microenvironment (TME), exogenous supplementation with PUFAs along with inhibited lipid droplets (LDs) biogenesis rendered cancer cells sensitive to ferroptosis [48]. Similarly, the PUFAs α-eleostearic acid and dihomo-γ-linolenic acid induce ferroptosis and suppress tumor growth and metastasis [49, 50]. Evidence delineating the role played by altered lipid metabolism in different aspects of cancer phenotype acquisition has been reviewed by Marteinn et al. [51], suggesting that targeting lipid metabolism-related ferroptosis may open a promising window for malignant cancer treatment.

Molecular dynamics studies have revealed that increased lipid peroxidation directly enhances membrane permeability, changes membrane shape and curvature, and promotes increased cell accessibility to oxidants, eventually causing cell death [52]. Intracellular lipid peroxidation can be driven non-enzymatically by iron and reactive oxygen/nitrogen species, or enzymatically through specific lipid-oxidizing enzymes. The enzymes, involved in lipid peroxidation include acyl-CoA synthetase long-chain family member 4 (ACSL4) [53], iron-dependent LOXs [54] and cytochrome P450 oxidoreductase [55]. Acyl-CoA synthetase long-chain family member 4 (ACSL4), a key ferroptosis driver, preferentially ligates long-chain PUFAs such as AA (20:4) and AdA (22:4), enabling their esterification into phospholipids by lysophosphatidylcholine acyltransferases (LPCATs). A recent report demonstrated that ACSL4 was phosphorylated and activated by protein kinase C-βⅡ (PKCβⅡ), which sensed the initial lipid peroxides and amplified the lipid peroxidation linked to ferroptosis [56]. Consistent with these findings, genetic deletion of ACSL4 generated monounsaturated fatty acyl (MUFA) tails in PLs, which led to cell proliferation under conditions of *GPX4* knockout (KO) and protected cells from ferroptosis [53]. Furthermore, ACSL4 and LPCAT3 promote GPX4 inhibition‐induced ferroptosis in KBM7 cells [57]. This mechanism helps explain the ferroptosis sensitivity observed in certain therapy-resistant cancers, including mesenchymal-state cells and clear-cell renal carcinoma [6, 58]. Ferroptosis can also occur independently of ACSL4, as demonstrated in p53-ALOX12 axis-mediated tumor suppression, where ACSL4 is dispensable for ferroptosis induction [59]. However, whether other ACSL family members are involved in PL esterification by targeting ALOX12 during ferroptosis remains to be investigated.

Lipoxygenases (LOXs) are another key class of lipid-oxidizing enzymes that directly oxygenate PUFAs in membranes through iron-independent dioxygenation, thereby promoting ferroptosis [54]. *Alox15* KO and certain pharmacological inhibitors of LOX action prevented ferroptosis in mouse embryonic fibroblasts (MEFs) [60], and the LOX inhibitor baicalein protected mice from ischemic brain injury [61]. However, silencing *Alox15* expression in mice with a *Gpx4*-KO background did not prevent ferroptosis [62]. These data suggest that alternative mechanisms may compensate for ALOX15 inactivation because LOX inhibitors are radical-trapping antioxidants (RTAs) that impede ferroptosis. Hence, LOXs may not be key drivers of ferroptosis in some contexts but can be involved in initiation and propagation of lipid peroxidative damage in other contexts [5]. Consistent with this conclusion, *Alox15* or *Alox12* deficiency exerted a protective effect against neurodegeneration or cancer by suppressing ferroptosis in specific mouse models [59]. Further investigations into which specific LOXs member under what diseases contexts that is most relevant to ferroptosis are required to be answered. Beyond the context-dependent role of LOXs, cytochrome P450 oxidoreductase (POR) has emerged as a key ferroptosis mediator in various cancer cells [55]. In addition to POR, the oxidoreductase CYB5R1 contributes to lipid peroxidation and membrane damage, thereby inducing ferroptosis by transferring electrons from NAD(P)H to oxygen, producing hydroxyl radicals [63]. Ferroptosis can also be triggered independently of ACSL4 under conditions of ROS accumulation, for example, pleckstrin homology-like domain family A member 2 (PHLDA2) promotes phosphatidic acid peroxidation in tumors [64]. These recent findings offer novel ideas for targeting cytochrome P450 and PHLDA2 to induce ferroptosis for cancer therapy. In the acidic tumor microenvironment, n-3 and n-6 PUFAs accumulate beyond lipid-droplet storage capacity, driving lipid peroxidation and ferroptosis. This explains why dietary n-3 long-chain PUFAs exert enhanced antitumor effects even when diacylglycerol acyltransferase (DGAT) is inhibited [65]. Furthermore, in mesenchymal-type gastric cancer (GC) cells, the expression of elongation of very long-chain fatty acid protein 5 (ELOVL5) and fatty acid desaturase 1 (FADS1) was upregulated, maintaining intracellular levels of AA and AdA and leading to ferroptosis sensitization [66]. Recent work has revealed that peroxisomes contribute to ferroptosis initiation through pathways such as the FAR1-ether lipid-TMEM189-TMEM164 axis and the peroxisome-ether-phospholipid axis, positioning peroxisomes as attractive targets for ferroptosis-based anticancer strategies [67, 68] and peroxisome-ether-PL axis [69], highlighting peroxisomes as promising druggable targets for anticancer therapy based on ferroptosis.

Beyond the mechanisms that trigger lipid peroxidation, several protective pathways modulate lipid metabolism to suppress ferroptosis. The mevalonate pathway plays an important role in intracellular lipid metabolism and can be blocked by statins targeting hydroxymethylglutaryl-CoA reductase (HMGCR), which leads to ferroptosis [70]. Notably, another lipid metabolism pathway, cyclohydrolase-1 GCH1-BH4, also regulates lipid peroxidation by increasing BH4 production and CoQ10 levels [71]. Targeting GCH1-BH4 can be an effective way to sensitize cancer cells to ferroptosis induction. Additional negative regulators, such as phospholipase A2 (PLA2) and lysophosphatidylserine lipase ABHD12, work together to exclude damaged fatty acyl chains from incorporation into the cancer cell membrane [72]. DECR1, the rate-limiting enzyme in PUFA oxidation, suppresses lipid peroxidation and acts as a negative regulator of ferroptosis in prostate tumors [73]. Moreover, a bifunctional protein, peroxiredoxin-6 (PRDX6), exhibits ferroptosis inhibition activity by inducing PLA2 and peroxidase activities [74]. Recent work identifies pyruvate dehydrogenase kinase 4 (PDK4) as a ferroptosis suppressor in pancreatic cancer cells, where it inhibits pyruvate oxidation-dependent fatty acid synthesis [75]. This finding highlights metabolic rewiring as a potential target for overcoming ferroptosis resistance. In addition, compared to PUFAs, which are sensitive to lipid peroxidation, MUFAs show resistance to lipid peroxidation, and therefore, exogenous MUFAs can be used to replace PUFAs [76]. Stearoyl-CoA desaturase (SCD) is required for MUFA biosynthesis because it converts saturated fatty acids into MUFAs. In GC, high expression of SCD1 promoted tumor growth and migration by inhibiting ferroptosis and altering cancer stemness [77]. Similar protective roles of SCD1 have been observed in diverse cancers, including KRAS-mutant lung cancer [78], lymph-metastasized melanoma [79] and ovarian cancer cells [80]. Similarly, endogenous ether lipids protected fibrosarcoma HT-1080N cells from ferroptosis by antagonizing lipid-ROS propagation [50]. Recently, the roles played by various forms of cholesterol involved in tumorigenesis have been regularly reported, leading to a new understanding of the physiological functions of ferroptosis in cancer suppression. 27-Hydroxycholesterol (27HC) has been demonstrated to enhance the tumorigenic and metastatic capacity of multiple cancer cells by promoting resistance to GPX4-dependent ferroptosis [81]. As an intermediate metabolite of cholesterol, 7-dehydrocholesterol (7-DHC) acts as ferroptosis suppressor through inhibiting phospholipid autoxidation of plasma and mitochondria membranes [82]. Independently, Conrad et al. reported that endogenous 7-DHC acts as a potent ferroptosis suppressor in cancer cells, suggesting that 7-DHC accumulation can enable ferroptosis evasion in certain contexts [83]. Moreover, other newly defined factors are effective to prevent ferroptosis through changing phospholipid metabolism or scavenging lipid radicals, there negative regulators of ferroptosis include tumor suppressor cyclin-dependent kinase inhibitor 2 A (CDKN2A) [84], lipid flippase solute carrier family 47 member 1 (SLC47A1) [85], lysophosphatidylcholine acyltransferase 1 (LPCAT1) [86] and hydropolysulfides (GSSxH) [87].

Given that ferroptosis is driven by PL peroxidation, a long-standing question remains unanswered: what role, if any, does oxygen concentration play in this process? Notably, under hypoxia, intracellular antioxidant capacity is overwhelmed, driving the activation of HIFs. A recent study indicated that HIF2α was critical for the high sensitivity of clear cell carcinoma (CCC) cells to GPX4 inhibitor-induced ferroptosis and that this sensitivity was driven by HIF2α-related LD-associated protein (HILPDA)-induced enrichment of polyunsaturated lipids [58]. Based on studies of hypoxia-related signaling, several small-molecule compounds have been tested in a cancer therapy strategy in clinical trials, including 2-methoxyoestradiol (NCT00030095), BAY 87–2243 (NCT01297530), PX-478 (NCT00522652) and PT2385 [88]. Moreover, other metabolites or metabolic enzymes involved in the intracellular redox process have also been found to function as ferroptosis regulators in malignant cells. For example, pyruvate metabolism, a major source of superoxide in mitochondria, supported the ferroptosis of HT1080 cells [89]. In contrast, the aldehyde dehydrogenase 3a2 (ALDH3A2) enzyme oxidized LC aliphatic aldehydes to protect acute myeloid leukemia (AML) cells from ferroptosis [90]. Similarly, aldo–keto reductases (AKRs) confer ferroptosis resistance by degrading 12/15-LOX-generated lipid peroxides [91]. Furthermore, in hypoxic solid tumors, carbonic anhydrase IX (CAIX) regulated redox homeostasis with iron-sulfur cluster enzyme (NFS1), inducing ferroptosis resistance [92]. Moreover, cytosolic aspartate amino transaminase (GOT1) has been recently found to promote pancreatic cancer cell ferroptosis via the regulation of mitochondrial metabolism and labile iron availability [93]. Concerning that some cancers possess ferroptosis suppression capability, cellular surveillance system function critical role to inhibit ferroptosis. Recently, membrane bound O-acyltransferase domain containing 2 (MBOAT2) and MBOAT1 have been newly identified as a GPX4/FSP1-independent ferroptosis suppressors, which increase PE-MUFA and decrease PE-PUFA content in sex hormones- dependent manner in prostate cancer cells and breast cancer cells [94]. Additionally, in human renal carcinoma cells (RCCs), the metazoan SpoT homolog 1 (MESH1) gene has been found to be a ferroptotic regulator that decreases cytosolic levels of NADPH, and MESH1 depletion promoted ferroptosis [95]. Interestingly, microsomal glutathione S-transferase 1 (MGST1) has been discovered to be a negative regulator of ferroptosis in human pancreatic cancer cells; specifically, MGST1 inhibited ALOX5 activity and prevented oxidative damage [96]. Similarly, in human basal-like breast cancer (BLBC) cells, increased expression of the transsulfuration enzyme cystathionine β-synthetase (CBS) conferred protection against oxidative stress and ferroptosis by realigning cellular cysteine function persulfidation [97]. In gallbladder cancer, sirtuin 3 (SIRT3) suppressed AKT-dependent mitochondrial metabolism, causing ferroptosis and tumor growth suppression [98].

**Fig. 2:**
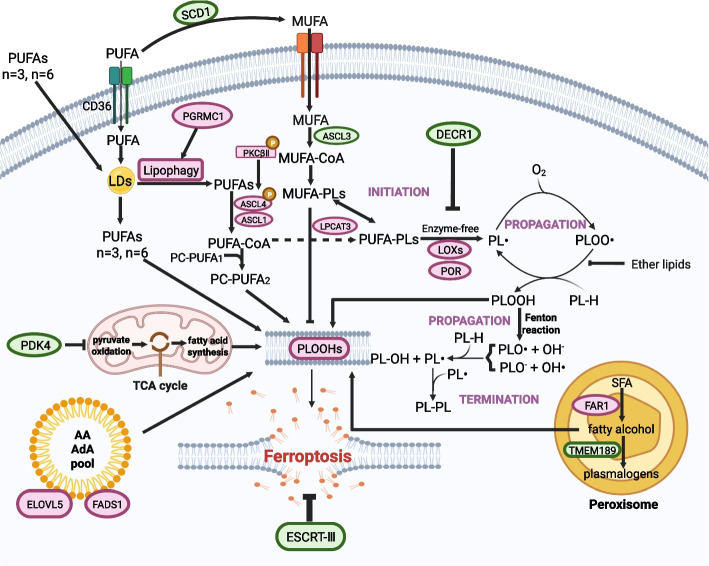
Regulation of lipid peroxidation triggers ferroptosis in cancer cells. Ferroptosis induction depends on lipid peroxidation, which can be initiated via enzymatic and nonenzymatic mechanisms, which also affect the ferroptotic rate and termination. Extracellular polyunsaturated fatty acid (PUFA) intake or lipophagy-mediated fatty acid release causes the accumulation of free fatty acids, which triggers a chain reaction mediated by long-chain fatty acid-CoA ligase 4 (ACSL4) and lysophospholipid acyltransferase 5 (LPCAT3) to form PUFA-containing phospholipids (PUFA-PLs). PUFA-PLs or PC-PUFA are used for the initiation of lipid peroxidation driven by the Fenton reaction or a lipoxygenase (LOX)/cytochrome P450 oxidoreductase (POR), resulting in the generation of PC/PL hydroperoxides (PC/PLOOHs). After their initial production, PC/PLOOHs are either rapidly cleared by glutathione peroxidase 4 (GPX4) or are subjected to the Fenton reaction and lipoxygenases, which further amplify PC/PLOOH production. Excessive PC/PLOOHs directly damage cell membranes and causes *nercosis-like* cell death. Lipid peroxidation can be terminated through a reductase system or radical-trapping antioxidants (RTAs). In addition, the tricarboxylic acid (TCA) cycle and peroxisomes, as well as arachidonic acid (AA) accumulation contribute to the lipid peroxidation and ferroptosis of cancer cells. In contrast, monounsaturated fatty acids (MUFAs) prevent the lipid peroxidation cascade reaction and play an antiferroptotic role, although the mechanisms are not yet understood. Similarly, endosomal-sorting complex required for transport-III (ESCRT-III) protects cell membranes from lipid peroxidation and ferroptosis

In summary, cancer cells need abundant PUFAs and few MUFAs to undergo ferroptosis. However, the mechanisms by which cells selectively peroxidize PUFAs over MUFAs, and how MUFAs confer protection, require further investigation. Targeting these key lipid metabolic regulators to induce lipid peroxidation represents a promising strategy for eliminating cancer cells (Fig. 2). However, the efficacy of ferroptosis-inducing agents is often limited by adaptive responses in cancer cells, such as genetic mutations and epigenetic alterations that enhance antioxidant defenses and suppress lipid peroxidation, thereby reducing ferroptosis sensitivity [5]. Hence, whether a given cancer cell is susceptible or resistant to ferroptosis under certain conditions is determined by the genetic background and metabolic features of lipid components. Therefore, the genomic and metabolic profile of a tumor should be considered when applying ferroptosis-based therapies.

### Antioxidant metabolism and ferroptosis

Glutathione (GSH), the most abundant non-protein thiol in mammalian cells, stands at the crossroads of cellular redox homeostasis and ferroptosis regulation. Its tripeptide structure (γ‑Glu‑Cys‑Gly) enables versatile antioxidant functions, primarily by serving as an essential co‑factor for glutathione peroxidase 4 (GPX4). GPX4 utilizes GSH to reduce toxic lipid hydroperoxides (LOOH) to harmless lipid alcohols (LOH), thereby halting the peroxidative chain reactions that drive ferroptosis. Consequently, the intracellular GSH pool directly determines cellular susceptibility to ferroptosis. Cancer cells, often exposed to high intrinsic oxidative stress due to accelerated metabolism and therapeutic insults, frequently upregulate GSH synthesis and regeneration pathways to reinforce their antioxidant defense [99]. This adaptive response not only supports tumor survival and proliferation but also confers resistance to conventional therapies, including chemotherapy and radiotherapy, which often rely on ROS generation to kill cancer cells [100]. Thus, targeting GSH metabolism to reactivate ferroptosis has emerged as a promising strategy to overcome therapy resistance in oncology.

The regulation of GSH in cancer is multifaceted, involving biosynthesis, utilization, and recycling. GSH synthesis depends critically on cysteine availability, which is primarily imported via the cystine/glutamate antiporter system Xc − (composed of SLC7A11 and SLC3A2) [101]. Once inside the cell, cystine is reduced to cysteine, the rate‑limiting substrate for GSH synthesis catalyzed by glutamate‑cysteine ligase (GCL) and glutathione synthetase (GSS) [102]. The GSH‑GPX4 axis is further supported by glutathione‑disulfide reductase (GSR), which regenerates GSH from its oxidized form (GSSG) using NADPH. In many cancers, oncogenic signaling pathways such as NRF2 (NFE2L2) are constitutively activated, leading to transcriptional upregulation of SLC7A11, GCL, and other antioxidant genes, thereby elevating GSH levels and ferroptosis resistance [103]. Conversely, tumor suppressors like p53 can inhibit SLC7A11 expression or promote pro‑ferroptotic metabolic shifts, rendering cells more vulnerable to lipid peroxidation [104]. Beyond its canonical role in GPX4 activity, GSH also modulates ferroptosis through indirect mechanisms: it chelates labile iron to suppress Fenton chemistry, maintains the activity of other antioxidant enzymes, and directly scavenges ROS and lipid peroxidation‑derived electrophiles [105]. Moreover, post‑translational modifications like S‑glutathionylation dynamically regulate the function of key proteins involved in iron metabolism, redox sensing, and death signaling, adding another layer of complexity to GSH‑mediated ferroptosis control [106].

Exploiting the dependency of cancer cells on GSH for ferroptosis resistance has inspired numerous therapeutic approaches. Direct inhibition of system Xc − depletes cysteine, impairing GSH synthesis and triggering ferroptosis [107] Similarly, small molecules that covalently bind to GSH or inhibit GSR activity can disrupt the redox cycle and amplify lipid peroxidation. More selective strategies target the stabilization or activity of GPX4; for instance, RSL3 and other GPX4 inhibitors directly inactivate the enzyme, bypassing upstream GSH synthesis [108]. In recent years, nanotechnology‑enabled platforms have further refined GSH‑targeting therapies. Smart nanosystems designed to release ferroptosis inducers in response to the tumor microenvironment can concurrently deplete GSH via Fenton‑like reactions or thiol‑consuming cascades, thereby achieving synergistic ferroptosis induction [109]. Importantly, combining GSH‑depleting agents with conventional therapies or with immune checkpoint inhibitors has shown promising pre‑clinical results across various cancer types by overcoming redox‑adapted resistance mechanisms and stimulating anti‑tumor immunity [110]. However, challenges remain, such as tumor heterogeneity in GSH metabolism, compensatory activation of alternative antioxidant pathways, and the dual role of GSH in both promoting survival and modulating immune cell function [111]. Future research must elucidate the organelle‑specific dynamics of GSH pools, define metabolic vulnerabilities in different tumor contexts, and develop biomarker‑guided combination regimens to harness ferroptosis for clinical benefit without inducing systemic toxicity [112].

### Emerging regulators and signaling pathways

**Fig. 3:**
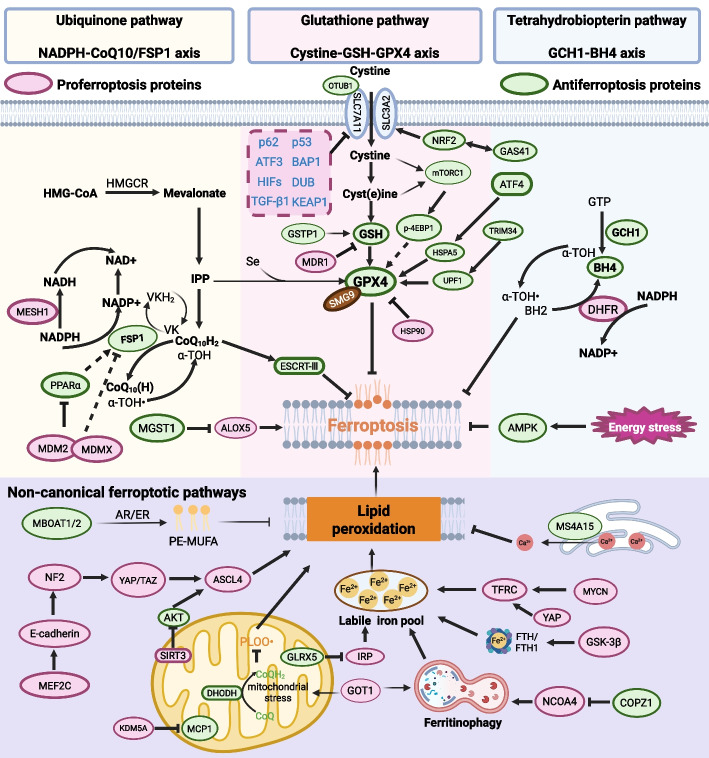
Signaling pathways in ferroptosis-based antitumor therapy. Ferroptosis induction is driven by excessive iron-dependent lipid peroxidation, which is elaborately regulated in cancer cells through disruption of the balance between the generation and elimination of lipid peroxides. The best characterized ferroptosis signaling pathways determine the abundance of antioxidants and scavenge lipid peroxides: the glutathione pathway (cystine-GSH-GPX4 axis), ubiquinone pathway (NADPH-CoQ10/FSP1 axis) and tetrahydrobiopterin pathway (GCH1-BH4 axis), which are particularly important for lipid peroxide elimination in cancer cells. The dysregulation of these three pathways contributes to excessive lipid ROS and lethal ferroptosis in tumor cells. In addition, noncanonical signaling pathways play important roles by regulating iron overload, lipid metabolism and energy metabolism, subsequently leading to the ferroptotic death of cancer cells

The molecular regulatory mechanism of ferroptosis is complicated because molecules related to iron metabolism, lipid peroxidation, redox homeostasis and energy metabolism can drive ferroptosis. In this section, we focus on signaling pathways that suppress tumors, highlighting key molecules and regulators that induce ferroptosis and represent promising therapeutic targets for refractory cancers (Fig. 3). Given the rapid advances of cancer biology, at least three canonical signaling pathways and several non-canonical pathways that regulate the sensitivity of cancer cells to ferroptosis have been identified.

#### Xc⁻-GSH-GPX4

System Xc − is composed of SLC7A11 and SLC3A2 and plays a key role as a cystine/glutamate antiporter that exchanges extracellular cystine for glutamate, cystine uptake results in enhanced formation of GSH, which in turn attenuates oxidative stress in cancer cells. In multiple types of human tumor cells, SLC7A11 has been found to be overexpressed and has been negatively correlated with patient survival [113]. When SLC7A11 expression was knocked out in tumor cells, tumor growth was abrogated in several mouse models [114, 115]. Because *SLC7A11*-KO mice were healthy and fertile, SLC7A11 is a promising target for the development of FINs, such as erastin and sorafenib (SRF), as discussed in the following therapy section. Furthermore, several proteins and molecules determine the vulnerability of cancer cells to ferroptosis by targeting SLC7A11. A recent report indicated that activating transcription factor 3 (ATF3) bound to the promoter of SLC7A11 and decreased the expression of SLC7A11, thereby inducing ferroptosis in a p53-independent manner [116]. In contrast, the Ub hydrolase OTUB1 was found to be a negative regulator of ferroptosis because it stabilized SLC7A11 in cancer cells [117]. GPX4 plays a central role in ferroptosis due to its ability to abrogate lipid peroxidation by catalyzing the reduction of PLOOHs. The expression and activity of GPX4 in ferroptosis relies on GSH expression and selenium [118]. Three amino acids, cysteine, glycine and glutamic acid, are precursors of GSH, limited mainly by cysteine availability. Notably, cysteine can be produced from extracellular cystine in a SLC7A11-dependent manner. Thus, the SLC7A11-GSH-GPX4 axis controls the sensitivity of cells to ferroptosis induction. Because GPX4 is a selenoprotein, the abundance of intracellular selenium determines the sensitivity of cancer cells to ferroptosis to some extent [118]. Drug-tolerant persister cells have been found to be highly dependent on GPX4 function for survival and therefore highly susceptible to GPX4 activity inhibition [119]. Rapamycin complex 1 (mTORC1) has been found to be a negative regulator of ferroptosis because it promotes GPX4 synthesis, suggesting that the combination of mTORC1 inhibitors and FINs is a promising cancer therapy [120]. By promoting the degradation of GPX4, smaug protein 9 (SMG9) has been identified as a positive driver of ferroptosis in human cancer cells [121]. Moreover, a newly defined ubiquitin protease termed Usp8 (ubiquitin-specific protease 8) which deubiquitinates GPX4 and stabilizes GPX4, thereby limit ferroptotic cell death [122]. Similarly, tripartite motif-containing protein 34 (TRIM34) interacts with Up-frameshift 1 (UPF1) and promotes its ubiquitin-mediated degradation, as a result, GPX4 expression is elevated and ferroptosis is prevented [123]. By targeting GPX4, a series of chemicals, such as RSL-3, FIN56, ML210 and altretamine, have been reported to induce ferroptosis in vitro and in mouse models [124, 125]. Beyond direct GPX4 targeting, many regulators affect ferroptosis sensitivity by modulating the GSH-GPX4 axis, including HSPA5 [126] and mTORC1 [127]. However, knocking out GPX4 gene expression led to embryonic lethal mice [62], indicating undesired side effects need to be considered when seeking to discover ferroptosis inducers that directly target GPX4 for cancer suppression. Additionally, although GPX4 plays a crucial role in ferroptosis, certain cancer cell lines are resistant to ferroptosis caused by GPX4 inhibitors [53], suggesting that other GPX4-independent molecules may regulate ferroptosis in tumors. A recent defined E3 ligase SMAD-specific E3 ubiquitin protein ligase 2 (SMURF2) has been shown to independently of GPX4 degrade GSH S-transferase P1 (GSTP1), thus, the regulation of SMURF2/GSTP1 can sensitize cancer cells to ferroptotic death [128]. Therefore, effective tumor suppression via ferroptosis may require targeting both GPX4-dependent and GPX4-independent pathways, depending on the cellular context.

#### NADPH-FSP1-CoQ10

Recently, screening of ferroptosis-suppressing genes in two studies led to the discovery of a novel molecule named AIFM2 (renamed ferroptosis suppressor protein 1 (FSP1)), which regenerated CoQ10 production and inhibited ferroptosis in a GSH-GPX4-independent manner [129, 130]. Mechanistically, FSP1 catalyzes the reduction of CoQ10 to ubiquinol, a lipophilic radical-trapping antioxidant that neutralizes lipid peroxides directly or by regenerating α-tocopherol (α-TOH). Moreover, a novel mechanism of FSP1 activity against ferroptosis has been recently found by accelerating the activity of the ESCRT-III membrane repair system, which functions independent of the CoQ10 pathway [131]. Similar to the genetic depletion of *Slc7a11*, loss of *Fsp1* did not induce embryonic lethality or tissue damage [132], indicating a promising therapeutic window through which to target FSP1 in tumor cells. Furthermore, many cell lines among 860 studied malignant cell lines were found to express FSP1 abundantly, rendering *Fsp1* one of the most highly expressed genes related to GPX4 inhibitor resistance [129]. Additionally, the specific ferroptosis inducer iFSP1 was discovered, iFSP1 with RSL3 treatment showed a synergistic effect to cause ferroptosis [129]. Moreover, FSP1 can undergo liquid–liquid phase separation, a recently identified mechanism that suppresses tumor growth and enhances the efficacy of ferroptosis inducers in combination therapy [133]. Interestingly, FSP1 functions as a reductase to reduce vitamin K to its hydroquinone by which radicals are scavenged and further intercept ferroptosis of cancer cells, exhibiting that targeting non-canonical vitamin K cycle can be an effective anticancer strategy [134]. In summary, developing specific inhibitors of the FSP1-CoQ10 pathway, particularly for clinical evaluation in refractory tumors, represents a promising strategy to overcome therapy-resistant cancers.

#### GCH1-BH4

In addition to the FSP1-CoQ10 pathway, another GPX4-independent pathway is related to the gene GTP cyclohydrolase 1 (GCH1), which limits the biosynthesis of the metabolite tetrahydrobiopterin (BH4) via lipid remodeling [71]. BH4 is a powerful RTA that is necessarily regenerated by dihydrofolate reductase (DHFR). Moreover, BH4 may promote the generation of CoQ10 by converting phenylalanine into tyrosine, which is then translated into 4-OH-benzoate, which is a precursor of CoQ10 [71]. However, further studies with BH4 and GCH1^–^/^–^ mouse models need to be carried out to explore the role played by GCH1-BH4 in ferroptosis. The precise role of the GCH1-BH4 axis in ferroptosis warrants further investigation using BH4 supplementation and GCH1^–^/^–^ mouse models. Dihydroorotate dehydrogenase (DHODH) has recently been identified as a mitochondrial ferroptosis defense system that reduces ubiquinone to ubiquinol, thereby inhibiting ferroptosis. Targeting DHODH thus represents a potential therapeutic strategy [127].

#### Noncanonical signaling pathways

The critical ferroptotic regulators aforementioned can be modulated by multiple oncoproteins and tumor suppressors, which constitute a complicated signaling network. Here, we discuss the pivotal signaling molecules that induce tumor growth suppression by triggering ferroptosis independent of classical signaling pathways.

Early studies suggested that oncogenic *Ras* mutations were required for erastin- and RSL3-induced ferroptosis [135], but subsequent work has refuted this notion [124]. In fact, mutation of *Ras* family oncogenes is one of the most common events in human cancers. Moreover, lung adenocarcinoma cells with *K-Ras* mutation were sensitive to ferroptosis induced by SLC7A11 inhibitors [136], and NSCLC-derived cells with mutation in the upstream RAS molecule exhibited susceptibility to ferroptosis [137]. *Ras* mutant signaling can influence ferroptosis sensitivity by regulating the expression of iron metabolism genes such as *TFRC, FTH1,* and *FTL* [135]. However, results that reportedly indicate that *Ras* mutation decreased the ferroptosis sensitivity of rhabdomyosarcoma-derived cells (RMS13) have been inconsistent [138], suggesting that a complex mechanism, which may be context-dependent and related to the genetic characteristics of different cancer cells. In addition to *Ras*, the transcriptional regulation of tumor suppressor p53 expression plays a key role in connecting tumor growth suppression and ferroptosis [139]. Mechanistically, p53 may promote cancer cell vulnerability to ferroptosis by inhibiting SLC7A11 expression [139], and loss of the acetylation site at K98 has been shown to abolish the ferroptosis-inducing function of p53 [140]. Moreover, p53 also regulates ferroptosis through downstream targets including SAT1, PTGS2, and GLS2 [124, 141]. Other findings revealed that ALOX12 plays a critical role in p53-mediated ferroptosis, with SLC7A11 directly interacting with ALOX12 to decreased ALOX12 activity and tumor cell proliferation [59]. Recently, poly ADP-ribose polymerase (PARP) inhibition has been shown to decrease SLC7A11 expression in a p53-dependent manner, inducing ferroptosis in BRCA-proficient ovarian cancer [142]. Furthermore, the direct and indirect targets of p53, including CDK, RB, and E2F, contributed to the activation of ferroptosis in a context-dependent manner [143].

Conversely, p53 can also inhibit ferroptosis, for example, by activating the p21 pathway to attenuate ROS accumulation and glutathione depletion [144]. p53 also mitigated ferroptosis in human colorectal cancer cells by directly binding the dipeptidyl-peptidase 4 (DPP4) to suppress NOX-mediated lipid peroxidation [145]. Two negative regulators of p53, the homologous proteins MDM2 and MDMX, have been verified to facilitate ferroptosis with or without p53 expression in HT-1080 and SK-Hep1 cells where MDM2-MDMX inhibition enhanced PPARα and FSP1 activity [146]. Thus, the precise role and mechanisms of p53 in ferroptosis and tumor suppression, particularly in refractory cancers, require further investigation.

Another tumor suppressor, BRCA1-associated protein 1 (BAP1), can promote ferroptosis by inhibiting SLC7A11 expression [147]. BAP1 is frequently mutated or deleted in numerous human cancers [147]. The mechanism through which BAP1 leads to tumor growth has been proposed to be induction of H2A ubiquitination (H2Aub) of the SLC7A11 promoter [147]. In addition, fumarase plays an important role in the tricarboxylic acid (TCA) cycle by converting fumarate to malate, and is a tumor suppressor in leiomyoma and papillary renal cell carcinoma [148]. Similar to p53 and BAP1, fumarase sensitizes cells to ferroptosis in response to cysteine starvation. Notably, loss of fumarase confers resistance to ferroptosis by interfering with the TCA cycle, suggesting the key role of normal metabolic mitochondrial functions in ferroptosis [149]. Fumarase promotes ferroptosis in a context-dependent manner, which may account for its variable role as a ferroptosis inducer across different cancer types. By regulating the TCA cycle, mitochondrial pyruvate carrier 1 (MCP1) has been demonstrated to be a negative regulator of ferroptosis in erlotinib-resistant head and neck cancer (HNC) cells via the EMT and glutaminolysis [150]. In addition, recent studies indicated that the E-cadherin-NF2-Hippo-YAP/TAZ pathway was an alternative signaling pathway for the regulation of ferroptosis in cancer therapy [151]. As a transcriptional coregulator, YAP can target multiple ferroptosis molecules, including ACSL4 and transferrin receptor TfR1, and cancer cells vulnerable to ferroptosis have been found to mainly depend on the suppression of the Hippo pathway and activation of YAP.

Myocyte enhancer factor 2 C (MEF2C) may drive E-cadherin and merlin (NF2) expression in meningioma, and genetic depletion of MEF2C has been shown to potentiate erastin-induced ferroptosis [152]. Similarly, a recent report demonstrated that a homolog of YAP named TAZ promoted ferroptosis in epithelial ovarian cancer cells expressing TAZ but not YAP [153]. Because E-cadherin, NF2, and Hippo are tumor suppressors often mutated in cancer, their status may serve as biomarkers for predicting tumor responsiveness to TAZ-induced ferroptosis [5]. Another important molecule involved in ferroptosis is nuclear factor erythroid 2-related factor 2 (NRF2), which plays a critical role in mitigating ferroptosis through the regulation of iron metabolism and GSH generation [154]. Mechanistically, glioma-amplified sequence 41 (GAS41) enhances NRF2 transcriptional activity on chromatin, promoting glutathione metabolic genes and thereby inhibiting ferroptosis in NSCLC cells [155]. In addition, posttranslational modification determines the role played by NRF2, which is deubiquitinated by ubiquitin-specific-processing protease 11 (USP11), and stabilizes the NRF2-USP11 complex, subsequently regulating cell proliferation and ferroptosis [156]. The therapeutic potential of inhibiting NRF2 to induce ferroptosis and improve cancer therapy merits further preclinical and clinical evaluation.

## Crosstalk with other cell death modalities

**Fig. 4:**
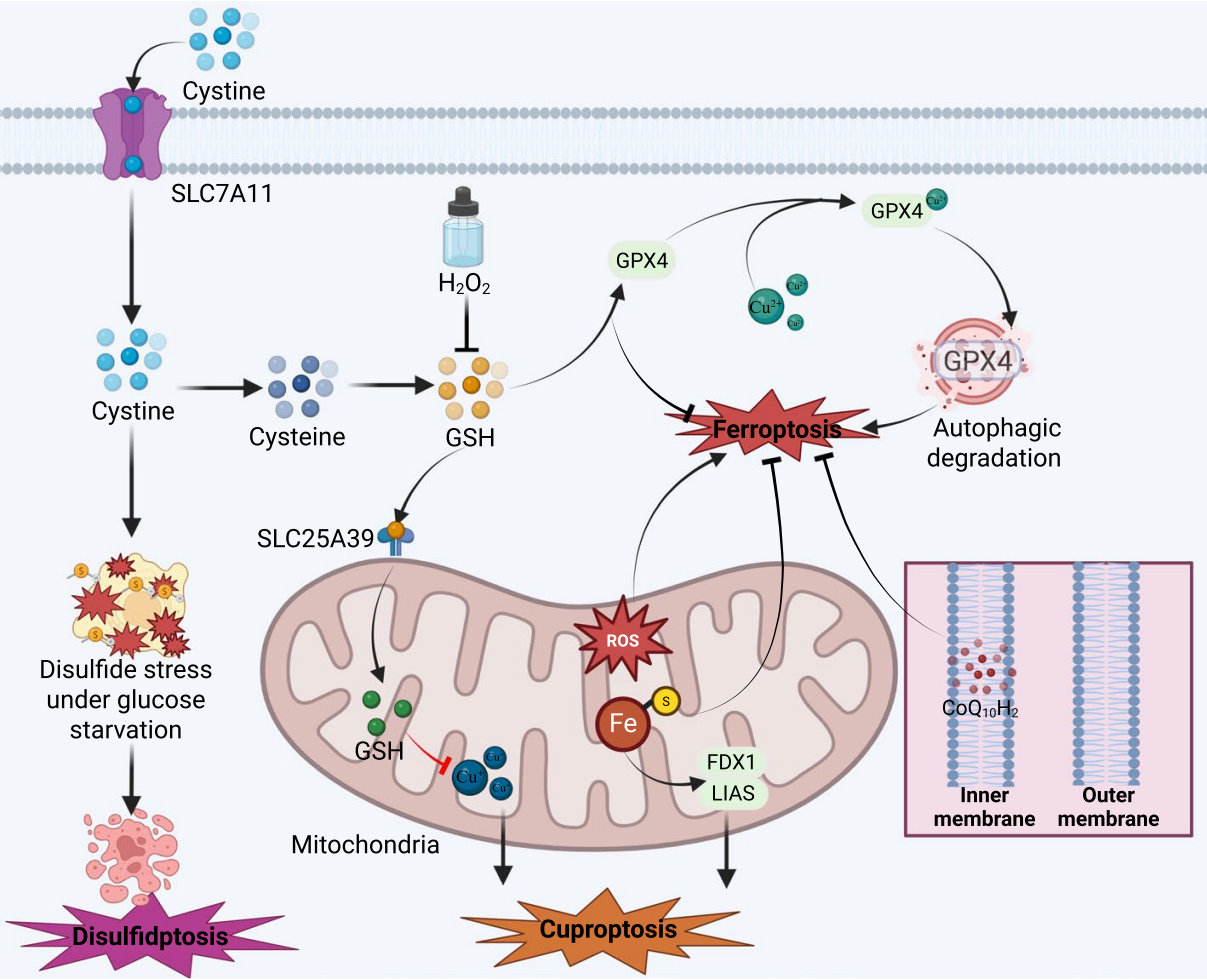
Crosstalk between ferroptosis with cuproptosis and disulfidptosis. Cystine import via SLC7A11 supplies intracellular cysteine for GSH synthesis, supporting GPX4-dependent detoxification of peroxides and thereby suppressing ferroptosis; however, in SLC7A11-high cells under glucose starvation, excessive cystine accumulation induces disulfide stress and triggers disulfidptosis, and H₂O₂ exposure can further deplete GSH, exacerbating redox imbalance. Mitochondria act as a central hub by shaping ROS production and iron-related oxidative stress and by supporting membrane antioxidant capacity (e.g., CoQ10H₂), which together influence ferroptosis sensitivity. In parallel, toxic accumulation of mitochondrial Cu^2^⁺ activates cuproptosis, with key regulators such as FDX1 and LIAS linking this pathway to mitochondrial metabolism, while GSH provides protection by chelating Cu^2^⁺. Moreover, Cu^2^⁺ can promote ferroptosis by facilitating autophagic degradation of GPX4, thereby weakening the GSH–GPX4 antioxidant defense and reinforcing the coupling between copper stress and ferroptotic cell death

Ferroptosis was originally described as a biochemically and morphologically distinct form of regulated cell death (RCD). However, accumulating evidence indicates extensive crosstalk between ferroptosis and other RCD modalities, including cuproptosis, disulfidptosis (Fig. 4) and autophagy-dependent cell death. These pathways share multiple upstream stress signals such as oxidative stress, mitochondrial dysfunction, and metabolic reprogramming, and often converge on common organelles and signalling hubs. Understanding these interactions is crucial for predicting therapeutic outcomes and designing effective combination strategies in cancer.

### Cuproptosis and ferroptosis

Cuproptosis is a recently identified copper-dependent form of regulated cell death that is mechanistically distinct from ferroptosis but shares an origin in metal dyshomeostasis and mitochondrial stress. The seminal work by Tsvetkov and colleagues demonstrated that targeted accumulation of Cu^2^⁺ in mitochondria triggers cell death by directly binding to lipoylated tricarboxylic acid (TCA) cycle enzymes, causing their aggregation and loss of iron–sulfur cluster proteins, ultimately leading to proteotoxic stress and mitochondrial failure [157]. Follow-up studies have further elaborated the cellular and molecular mechanisms of copper-induced death and formalised the concept of cuproptosis as a distinct RCD modality [158, 159]**.** In contrast to ferroptosis-which relies on iron-driven phospholipid peroxidation in PUFA-rich membranes-cuproptosis preferentially affects cells with high mitochondrial respiratory activity and abundant lipoylated TCA enzymes. Nevertheless, both ferroptosis and cuproptosis are tightly linked to cellular metabolism and trace metal homeostasis. Recent conceptual frameworks have proposed that different metabolic niches within tumors may be differentially susceptible to ferroptosis (in glycolytic, PUFA-rich cells) or cuproptosis (in oxidative, lipoylated TCA-dependent cells), suggesting that combined “metal-targeted”strategies exploiting both iron- and copper-dependent death pathways might provide therapeutic benefit in cancer [160].

### Disulfidptosis and ferroptosis

Disulfidptosis is another newly recognised form of regulated cell death, defined by lethal disulfide stress and collapse of the actin cytoskeleton. It was first characterised in SLC7A11-high cancer cells subjected to glucose starvation, where excessive cystine import in the absence of sufficient NADPH leads to aberrant accumulation of intracellular disulfides and abnormal disulfide bonding of actin-associated proteins, resulting in F-actin disassembly and cell death [161]. Morphologically and mechanistically, disulfidptosis is distinct from apoptosis, necroptosis, pyroptosis and ferroptosis, yet it arises from the same redox and metabolic networks that control ferroptosis [162]. A central player is SLC7A11: its inhibition promotes ferroptosis by limiting cystine uptake and glutathione synthesis, whereas its overexpression protects against ferroptosis but predisposes cells to disulfidptosis under conditions of glucose deprivation and NADPH shortage [163]. Thus, the same transporter can either safeguard cells from ferroptosis or drive disulfidptosis, depending on nutrient context and redox balance. Recent analyses further suggest that lipid peroxidation and disulfide stress may coexist in highly oxidative tumors, and that targeting NADPH-producing pathways or cytoskeletal redox homeostasis could shift the balance between ferroptosis and disulfidptosis as two complementary strategies for killing cancer cells.

### Autophagy and ferroptosis

**Fig. 5:**
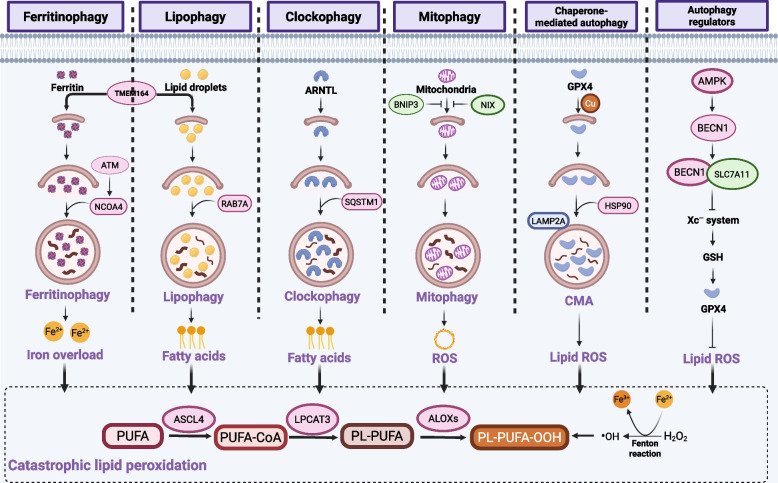
Selective autophagy-dependent ferroptosis in cancer therapy. Excessive autophagy contributes to ferroptotic induction in cancer cells. The known modalities of selective autophagy related to ferroptosis are ferritinophagy, lipophagy, clockophagy, mitophagy and chaperone-mediated autophagy, which provoke lipid peroxidation and ferroptosis. NCOA4-mediated ferritin degradation termed ferritinophagy induces free irons overload and ferroptosis. RAB7A-mediated lipid droplets degradation leads to lipophagy, resulting to fatty acids accumulation in ferroptosis. SQSTM1-mediated clockophagy degrades ARNTL and promotes lipid peroxidation in ferroptosis. Excessive mitophagy leads to the generation of lipid ROS and subsequent ferroptosis. Chaperone-mediated autophagy dependent on HSP90-mediated LAMP2A stability results in lipid peroxidation and ferroptosis. In addition, autophagy regulators such as BECN1-mediated Xc^−^ system inhibition contributes to GPX4 depletion and lipid peroxidation in ferroptosis. Thus, the selective autophagy can be an avenue for the induction of excessive lipid peroxidation and ferroptosis in cancer therapeutics

As a highly conserved degradation system, autophagy plays a key role in malignant tumors and can be leveraged as an important target in antitumor therapy. Recent increasing evidence has indicated that ferroptosis may depend on autophagy due to the elevation of intracellular iron, free fatty acids and other positive regulators of ferroptosis released from autolysosome [164]. Although ferroptosis can happen without autophagy, which may did not regulate iron release, lipid peroxidation and radicals generation in certain cancer contexts [165]. The induction of autophagy-dependent ferroptosis and the regulation of autophagy may be potential therapeutic strategies in fighting against neoplastic diseases [166]. Herein, we focus on selective autophagy in promoting cancer cells ferroptosis (Fig. 5).

Unlike macroautophagy (hereafter referred to as autophagy), selective autophagy precisely releases substrates required for iron-dependent lipid peroxidation, thereby promoting ferroptosis. The best characterized forms of selective autophagy related to the ferroptosis of cancer cells are ferritinophagy, lipophagy, mitophagy, clockophagy and chaperone-mediated autophagy (CMA). Ferritinophagy, namely, autophagic degradation of ferritin, drives ferroptosis via the accumulation of free intracellular iron in several types of tumor cells. Mechanistically, NCOA4 binds to low levels of FTH1 in autophagosomes and recruits ferritin for lysosomal degradation, resulting in elevated levels of intracellular irons, which catalyzes Fenton reaction, accounting for cancer cells ferroptosis [22]. During this process, a Ser/Thr protein kinase ATM dominated the elevation of labile iron pool by phosphorylating NCOA4 and promoting ferritinophagy [167]. In contrast, a negative regulator of ferritinophagy-dependent ferroptosis has been identified in HNC cells: poly(rC)-binding protein 1 (PCBP1) [168]. In addition, vitamin C has been demonstrated to induce ferritin phagocytosis and subsequent ferritin degradation and thus led to ferroptosis in anaplastic thyroid cancer (ATC) cells [169]. Other signaling pathway results in ferritin degradation, such as the autophagy-independent lysosomal degradation, is warranted for the therapeutic leverage of ferroptosis. Furthermore, because of the lipid requirement in lipid peroxidation leading to ferroptosis, lipophagy is important to ferroptosis; specifically, the autophagic digestion of LDs and increased levels of intracellular free fatty acids, which subsequently lead to ferroptosis [170]. In this process, the small GTPase RAB7A acts as a lipid‑droplet cargo receptor that facilitates LD delivery to multivesicular bodies and lysosomes. In contrast, increased lipid storage inhibited RSL3-induced ferroptosis, suggesting a negative relationship between LDs and ferroptosis. In HNC cells, the expression of PGRMC1 increased the fatty acid oxidation and ferroptosis rate via lipophagy in paclitaxel-tolerant persister cancer cells [46]. These findings indicate that the balance between lipid storage and degradation determines the ferroptosis sensitivity. In addition, mitophagy maintains the quantity and quality of mitochondria, which play a complicated role in ferroptotic death. Studies indicated that the mitochondrial complex I inhibitor BAY87-2243 induced mitophagy in melanoma cells [171], and the expression of HO-1 mediated IκBα inhibitor-induced ferroptosis [172]. Conversely, mitophagy can also remove damaged mitochondria and thereby protect cells from ferroptosis, as observed in HT-1080 cells [149]. The mitophagy regulators BNIP3 and NIX suppress ferroptosis by inducing mitophagy and reducing mitochondrial ROS [173]. Dual knockout of BNIP3 and NIX may therefore be a strategy to induce ferroptosis in cancer cells. These contradictory findings indicate the complex regulation of mitophagy during ferroptosis, suggesting that additional detailed insights are needed to elucidate the exact mechanisms in cancer.

Another form of selective autophagy, clockophagy, has been recently identified as the mechanism through which aryl hydrocarbon receptor nuclear translocator-like (ARNTL) is degraded by type-Ⅱ FINs [174]. ARNTL-degradation-associated clockophagy depends on the cargo receptor SQSTM1 and several ATG proteins and the blockade of HIF1A-dependent fatty acid uptake and lipid storage [174]. To date, how disrupted circadian rhythms affect ferroptosis sensitivity in cancer cells remains unclear. Moreover, chaperone-mediated autophagy (CMA) has been shown to play a key role in driving ferroptosis through the degradation of GPX4 in multiple cancer cell types, including pancreatic cancer cells [126] and breast cancer cells [175]. Mechanistically, HSP90 increases the stability of lysosomal-associated membrane protein 2 A (LAMP2A), which enhances GPX4 degradation and promotes ferroptosis [175]. Copper can promote GPX4 degradation via autophagy by directly binding to cysteine residues C107 and C148 on GPX4, enhancing its ubiquitination and subsequent Tax1 binding protein 1 (TAX1BP1)-mediated autophagic clearance, thereby driving ferroptosis [176]. Similarly, acid sphingomyelinase has been recently identified as a novel mediator in the autophagic degradation of GPX4 and ferroptosis induction in HT-1080 cells [177]. Autophagy-mediated GPX4 degradation has been identified in the Fin56-induced ferroptosis of bladder cancer cells, and the combined use of mTOR inhibitor with Fin56 exerted a synergistic effect to eliminate cancer cells [178]. Further understanding of CMA-mediated ferroptosis may provide a novel approach for developing precise cancer medicines. Identifying the key determinants of selective autophagy-dependent ferroptosis would facilitate its translation into anticancer therapies. Recent work indicates that transmembrane protein 164 (TMEM164) plays a central role in mediating the autophagic degradation of ferritin, lipid droplets, and GPX4 [179]. Therefore, activation or overexpression of TMEM164 may improve the survival of patients with pancreatic cancer through induction of autophagy-dependent ferroptosis. In addition to the aforementioned forms of selective autophagy involved in ferroptosis, reticulophagy and several other autophagy genes function as regulators during ferroptosis [21, 180]. Among these proteins, BECN1 has been identified as a novel SLC7A11/system Xc^—^-binding protein that facilitated ferroptosis [181]. Similarly, intracellular and extracellular HMGB1 can promote ferritinophagy, mitophagy, and general autophagy, leading to ferroptosis [21]. This suggests a broader functional interplay among HMGB1, autophagy, ferroptosis, and antitumor immunity that merits further investigation.

In conclusion, although certain modalities of selective autophagy play key roles in driving ferroptosis, the detailed mechanisms of autophagy-dependent ferroptosis require intensive study to advance our understanding. Finally, the development of small molecules targeting specific degradation pathways may facilitate the therapeutic leveraging of ferroptosis.

## Ferroptosis in cancer biology

The dysregulated metabolism and proliferative drive that define cancer cells also create unique metabolic vulnerabilities. Ferroptosis, an iron-dependent, lipid peroxidation-driven cell death pathway, has emerged as a critical frontier in cancer biology. Its importance in oncology arises from both the intrinsic susceptibility of many cancers to ferroptosis and the sophisticated adaptive mechanisms tumors employ to evade it. This section examines the dual nature of ferroptosis in oncology: the inherent cancer cell vulnerability that makes it a promising therapeutic target, the common strategies of ferroptosis evasion in cancer that contribute to tumor progression and therapy resistance, and the adaptive resistance mechanisms that can be acquired upon therapeutic pressure.

### Cancer cell vulnerability

Many cancer cells exhibit an inherent hypersensitivity to ferroptosis, a susceptibility rooted in their transformed metabolic state. This vulnerability results from multiple factors that collectively increase the intracellular risk of lethal lipid peroxidation. First, the high metabolic rate and mitochondrial dysfunction common in tumors lead to increased generation of reactive oxygen species (ROS), creating a pro-oxidant intracellular environment that primes membranes for peroxidation [182]. Oncogenic signaling pathways can exacerbate this state. For instance, mutant KRAS common in pancreatic and lung cancers drives proliferation while also upregulating iron import via transferrin receptor 1 (TFRC) and PUFA synthesis, thereby increasing both the catalyst iron and the peroxidizable lipid substrate for ferroptosis [183]. The composition of cellular membranes further dictates ferroptosis sensitivity. Cancer cells often upregulate the biosynthesis and incorporation of polyunsaturated fatty acids (PUFAs), such as arachidonic acid (AA) and adrenic acid (AdA), into membrane phospholipids. Enzymes such as acyl-CoA synthetase long-chain family member 4 (ACSL4) and lysophosphatidylcholine acyltransferase 3 (LPCAT3) esterify PUFAs into membrane phospholipids, providing substrates for lipoxygenases (LOXs) and cytochrome P450 oxidoreductase (POR) to initiate peroxidation [184]. Recent single-cell sequencing in breast and renal cancers identifies ACSL4 as a biomarker of ferroptosis sensitivity, with high ACSL4 expression correlating with improved responses to GPX4 inhibitors in preclinical models [185].

The therapeutic implication of this inherent vulnerability is profound. It suggests that triggering ferroptosis could be a “Achilles’ heel” strategy, selectively targeting cancer cells while sparing normal tissues with more robust antioxidant defenses. This is evidenced by the efficacy of compounds like erastin (which inhibits system xc⁻, depleting glutathione) and RSL3 (which directly inhibits GPX4) across a wide range of preclinical cancer models [100]. Moreover, this vulnerability provides a mechanistic basis for overcoming resistance to conventional therapies. Many chemotherapy drugs and radiotherapy act in part by generating ROS. Co-targeting the GSH or GPX4 antioxidant axis with ferroptosis inducers can therefore overcome redox adaptation and resensitize resistant tumors [186]. Recent clinical translation efforts are exploring pharmacodynamic biomarkers, such as measuring malondialdehyde (MDA) or 4-hydroxynonenal (4-HNE) in tumor biopsies, to validate target engagement in early-phase trials of ferroptosis-inducing agents [187].

### Ferroptosis evasion in cancer

Despite inherent vulnerabilities, successful tumor progression necessitates the development of robust defense mechanisms to evade ferroptosis. This evasion is a hallmark of advanced cancers and a key contributor to therapeutic resistance. The most prominent defense is the upregulation of the glutathione (GSH)-glutathione peroxidase 4 (GPX4) axis. Many cancers exhibit increased expression of the cystine/glutamate antiporter (system Xc⁻, SLC7A11) to enhance cysteine import for GSH synthesis. This is often driven by constitutive activation of the transcription factor NFE2-like BZIP transcription factor 2 (NRF2), resulting from somatic KEAP1 mutations or oncogenic signaling via KRAS, PI3K, or RAF [188]. Activated NRF2 transactivates a battery of antioxidant genes, including SLC7A11, GCLC (the rate-limiting enzyme in GSH synthesis), and GPX4 itself, creating a fortified antioxidant shield [189]. Metabolic rewiring can also generate endogenous anti-ferroptotic molecules. Elevated synthesis of 7-dehydrocholesterol (7-DHC) or squalene in certain lymphomas and other cancers directly scavenges lipid radicals, creating a robust barrier against ferroptosis [83].

Beyond the core GSH-GPX4 axis, tumors activate alternative, parallel pathways to detoxify lipid peroxides. The ferroptosis suppressor protein 1 (FSP1) has emerged as a major GPX4-independent resistance mechanism. FSP1 utilizes NAD(P)H to reduce coenzyme Q10 (CoQ10) to ubiquinol, a potent lipophilic antioxidant that halts the propagation of lipid peroxidation chains within membranes [94]. High FSP1 expression, driven by gene amplification or transcription factors such as E2F1, correlates with resistance to GPX4 inhibitors in lung, colon, and other cancers. Another key player is dihydroorotate dehydrogenase (DHODH), which, in mitochondria, also reduces CoQ10 to ubiquinol, providing organelle-specific protection [190]. Furthermore, the mevalonate pathway, which generates cholesterol and isoprenoids, supplies the substrate for CoQ10 biosynthesis, linking metabolic reprogramming directly to ferroptosis defense [191]. Inhibiting this pathway with statins or directly targeting FSP1 with small-molecule inhibitors can sensitize resistant cells to ferroptosis, and combination strategies are under active investigation [108].

The tumor microenvironment (TME) plays an active role in enabling ferroptosis evasion. Cancer-associated fibroblasts (CAFs) can secrete cysteine and other metabolites such as glutathione to support the antioxidant capacity of neighboring tumor cells, effectively functioning as a paracrine ferroptosis suppression system [192]. Additionally, hypoxia, a common feature of solid tumors, can stabilize Hypoxia-Inducible Factors (HIFs), which transcriptionally repress pathways that promote ferroptosis, such as iron uptake via TFRC, while promoting glycolytic metabolism that may alter lipid precursor availability [193]. The dense, fibrotic structure of some TMEs may also physically limit the diffusion of oxygen and peroxides, inadvertently slowing peroxidation kinetics. These microenvironmental adaptations underscore that ferroptosis evasion is not solely a cell-autonomous process but is supported by the entire tumor ecosystem, necessitating therapeutic strategies that also target matrix components.

### Resistance mechanisms

When therapeutic pressure is applied through ferroptosis-inducing agents, cancer cells can deploy both pre-existing and acquired resistance mechanisms to survive. A primary adaptive response is metabolic rewiring to bypass targeted pathways. For instance, upon inhibition of system xc⁻ by erastin or sulfasalazine, cells may activate alternative cysteine acquisition routes [194]. These include the transsulfuration pathway, where methionine is converted to cysteine via the CBS/CGL pathway, or enhanced macropinocytosis to scavenge extracellular proteins and release cysteine via lysosomal degradation [100]. Similarly, GPX4 inhibition can lead to the compensatory upregulation of parallel antioxidant systems, most notably FSP1 or DHODH, as discussed previously. This redundancy necessitates combination therapies that concurrently target multiple ferroptosis defense nodes, for example, jointly inhibiting GPX4 and FSP1.

Genetic and epigenetic alterations also underpin acquired resistance. Loss-of-function mutations in genes promoting ferroptosis such as ACSL4 or amplification/gain-of-function in suppressor genes (e.g., NFE2L2, FSP1) can be selected for under treatment pressure. Epigenetic regulation, including histone methylation and acetylation, dynamically controls the expression of ferroptosis-related genes [195]. For example, inhibition of histone methyltransferase G9a can suppress the expression of pro-ferroptotic genes and sensitize resistant pancreatic cancer cells [196]. Furthermore, post-translational modifications like ubiquitination and degradation regulate key players; the stability of GPX4 and SLC7A11 is controlled by various E3 ligases and deubiquitinases. The deubiquitinase USP8, for instance, stabilizes GPX4, and its inhibition promotes GPX4 degradation and ferroptosis sensitivity [122]. Manipulating these regulatory processes can reverse ferroptosis resistance in cancer. Resistance is further complicated by the dynamic and often paradoxical interplay between ferroptosis and the tumor immune microenvironment. While ferroptosis can release damage-associated molecular patterns (DAMPs) and oxidized lipids that stimulate dendritic cell maturation and antitumor T-cell responses via the STING pathway, it may also recruit immunosuppressive myeloid-derived suppressor cells (MDSCs) and upregulate immune-checkpoint molecules such as PD-L1 on surviving tumor cells [110, 111]. This creates a dual effect where inducing ferroptosis might initially aid immunity but subsequently foster an immunosuppressive niche that protects residual, resistant cells. Therefore, a major emerging resistance mechanism is the therapy-induced remodeling of the TME toward an immunosuppressive state. Overcoming this requires rationally sequenced combinations of ferroptosis inducers with immune checkpoint blockers, MDSC-targeting agents, or strategies that block the “don’t eat me” signal CD47 to enhance phagocytic clearance of ferroptotic cells [197]. A clear understanding of these complex, adaptive, and microenvironment-mediated resistance networks is essential for designing durable ferroptosis-based therapeutic regimens.

## Ferroptosis in different types of cancer

**Fig. 6:**
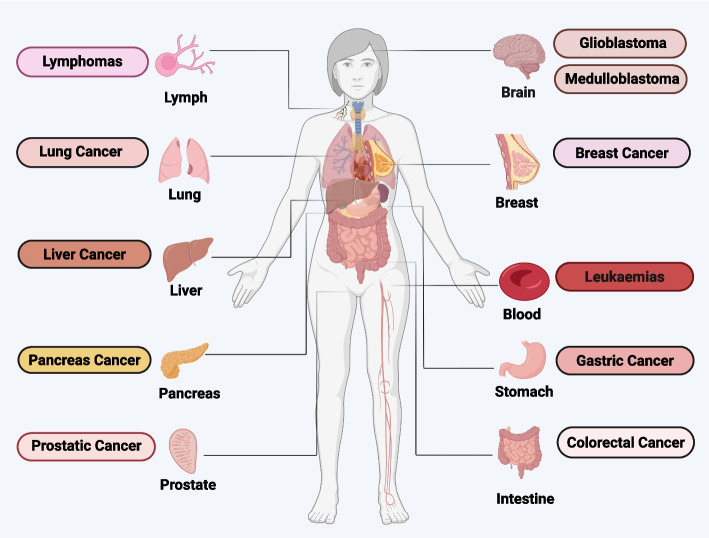
Ferroptosis in different types of cancer. This schematic summarizes the involvement and therapeutic relevance of ferroptosis across diverse human malignancies by mapping representative cancer types to their primary anatomical sites. The figure highlights ferroptosis-associated studies and potential ferroptosis-targeting strategies in hematological cancers (lymphomas and leukaemias) and in multiple solid tumors, including lung cancer, liver cancer, pancreatic cancer, prostate cancer, breast cancer, gastric cancer and colorectal cancer, as well as central nervous system tumors such as glioblastoma and medulloblastoma

Emerging evidence shows that ferroptosis is not uniform across cancer types; instead, susceptibility to ferroptosis is determined by lineage-specific patterns of iron handling, lipid metabolism, and antioxidant defense. In this section, we briefly summarise how these features converge on ferroptosis regulation in major tumor entities (Fig. 6).

### Lung cancers

Ferroptosis in lung cancer is closely intertwined with oncogenic redox adaptation and treatment response, and many lung tumor models exhibit a pronounced dependence on the System Xc⁻-GSH-GPX4 antioxidant axis that was established in the seminal ferroptosis literature [3, 5, 124]. In NSCLC, frequent activation of the KEAP1-NRF2 stress-response programme supports cystine uptake and lipid-peroxide detoxification, thereby conferring resistance to oxidative stress while simultaneously creating targetable ferroptosis-defense dependencies; for example, ferroptosis protection in LUAD has been linked to KEAP1-altered contexts and metabolic lipid rewiring [198, 199]. Consistently, pharmacological inhibition of System Xc⁻ or GPX4 can trigger ferroptosis and may resensitize resistant NSCLC cells to standard chemotherapy; notably, ferroptosis inducers (erastin, RSL3) were reported to synergize with docetaxel and overcome chemoresistance by promoting ferroptosis-associated redox collapse and lipid ROS accumulation [200]. Small-molecule or natural-product approaches can converge on GPX4 destabilization as well; bufotalin has been shown to induce ferroptosis in NSCLC cells by promoting GPX4 ubiquitination/degradation and enhancing lipid peroxidation [201]. Beyond tumor-intrinsic killing, ferroptosis regulation may also intersect with immune phenotypes in lung cancer: SLC7A11 has been linked to ferroptosis suppression alongside altered PD-L1 levels in lung adenocarcinoma, and ferroptosis-related transcript/lncRNA signatures have been explored for associations with immune infiltration and predicted immunotherapy responsiveness [202, 203]. In SCLC, preclinical evidence likewise supports strong ferroptosis sensitivity upon disruption of GPX4-centered defenses; an RRM2–PRDX6–GPX4 axis has been reported to suppress ferroptosis by inhibiting GPX4 ubiquitination and to reduce cisplatin sensitivity, nominating this pathway as a potential resistance node [204]. Overall, these findings motivate ferroptosis-based combination strategies as complements to existing chemo-immunotherapy regimens in refractory lung cancers, while highlighting the translational gap and the need for clinically tractable ferroptosis modulators.

### Brain tumors

Brain tumors, especially glioblastoma (GBM), exist in a state of persistent oxidative and metabolic stress, leading to upregulation of antioxidant and iron-handling programs. Paradoxically, these adaptations can render them dependent on ferroptosis-defense pathways, creating therapeutic vulnerabilities. In GBM, temozolomide (TMZ) has been reported to engage ferroptotic mechanisms in addition to DNA damage, including a DMT1-associated iron-import axis that increases iron loading, lipid peroxidation and ferroptosis markers, suggesting that reinforcing ferroptosis can enhance TMZ efficacy in resistant contexts [205]. Complementing this concept, direct GPX4 inhibition has shown TMZ-sensitizing activity in glioma models, with evidence from both subcutaneous and orthotopic settings supporting combined ferroptosis induction and TMZ treatment [206]. Mechanistically, ferroptosis resistance in GBM can also be sustained by iron-homeostasis and mitochondrial redox circuits: amplification of IRP1 signalling was shown to reverse TMZ resistance through an NFKB2-LCN2/FPN1-linked axis [207], and a PRR11-DHODH module has been implicated in ferroptosis and TMZ resistance [208]. In parallel, cell-state-resolved analyses indicate that specific glioblastoma states (e.g., quiescent/astrocyte-like populations) may be preferentially vulnerable to GPX4 blockade [209], reinforcing the idea that ferroptosis sensitivity is heterogeneous within GBM.

Beyond GBM, ferroptosis-relevant transcriptional programmes have been linked to medulloblastoma molecular subtypes and prognosis, supporting the plausibility of subtype-informed ferroptosis targeting in paediatric brain tumors [210]. Additional mechanistic work suggesting that perturbing mitochondrial Fe-S handling can potentiate cisplatin via ferroptosis has been reported, but key evidence is currently presented as meeting abstracts and/or preprints and should be interpreted accordingly [211]. Importantly, any ferroptosis-inducing strategy for brain tumors must be balanced against on-target toxicity in the CNS: conditional loss of neuronal GPX4 is sufficient to drive neurodegeneration and cognitive impairment [212], and neuronal physiology makes the brain particularly vulnerable to lipid-peroxidation-driven death [213].

### Gastrointestinal cancers

In colorectal cancer (CRC), patient tissues frequently display elevated SLC7A11 and GPX4, consistent with an antioxidant “buffer” that supports tumor progression and correlates with adverse outcome [214]. Mechanistically, CRC cells can actively suppress ferroptosis by stabilising GPX4: HSPA5 has been reported to bind GPX4 and slow its degradation, thereby restraining ferroptosis and promoting CRC growth [215]. Epigenetic regulation also intersects with this pathway; for example, HDAC3 modulates ferroptosis via an NRF2-GPX4 axis in CRC cells [216]. Beyond cancer-cell intrinsic defences, computational and experimental studies suggest that ferroptosis regulatory patterns in CRC associate with distinct tumor microenvironment states and [217], which may help stratify immunotherapy responsiveness.

In gastric cancer, ferroptosis sensitivity is likewise shaped by redox and lipid metabolic programs: SLC7A11 has been linked to malignant progression and ferroptosis regulation through PI3K/AKT signalling [218]. Targetable upstream regulators are emerging, exemplified by USP7 inhibition (via DHPO) triggering ferroptosis through destabilising stearoyl-CoA desaturase (SCD), with in vivo anti-tumor activity in orthotopic models [219]. Consistent with a broader immune-redox coupling, GPX4 downregulation in gastric cancer has been reported to restrain tumor growth alongside reduced M2 macrophage polarisation and increased CD8 + T cell infiltration [220]. Pancreatic ductal adenocarcinoma (PDAC) presents a particularly strong dependency on cyst(e)ine metabolism: genetic deletion of Slc7a11 or pharmacologic cysteine/cystine depletion (cyst(e)inase) can induce tumor-selective ferroptosis and suppress PDAC growth in genetically engineered mouse models [115]. Metabolic rewiring also creates actionable liabilities, GOT1 suppression has been shown to inhibit PDAC growth and render cells vulnerable to oxidative stress–driven death consistent with ferroptosis [93]. At the same time, PDAC biology highlights a critical nuance. Ferroptosis-associated damage (e.g., GPX4 loss or iron overload) can, in specific inflammatory contexts, promote tumor initiation via a STING (TMEM173)-dependent DNA-sensing pathway [221], underscoring that ferroptosis can be therapeutically beneficial yet biologically double-edged depending on timing and microenvironment.

### Hematological malignancies

Hematological cancers often sit at a precarious redox “tipping point”, where adaptations that sustain rapid proliferation (iron acquisition, PUFA-rich membrane remodeling, and heightened antioxidant buffering) can also create exploitable ferroptotic liabilities. In acute myeloid leukemia (AML), functional genetic screening identified ALDH3A2 as a selective metabolic dependency that detoxifies lipid-derived aldehydes and protects leukemic cells from oxidative death; notably, ALDH3A2 suppression shows synthetic lethality with GPX4 inhibition, linking aldehyde detoxification to the ferroptosis defense network [90]. Consistent with this axis, ALDH3A2 has also been reported to promote chemotherapy tolerance by supporting lipid metabolism and dampening Adriamycin-associated ferroptotic stress [90]. Systems-level analyses further suggest that ferroptosis-related transcriptional programs stratify AML into subgroups with distinct immune contexts and outcomes, supporting the value of ferroptosis signatures for prognosis and therapeutic hypothesis generation.

In acute lymphoblastic leukemia (ALL), a key mechanistic insight is that leukemic blasts can become selectively reliant on the GSH-GPX4 anti-ferroptotic axis because FSP1 is epigenetically silenced; restoring FSP1 increases resistance to GSH/GPX4-targeting stress, highlighting a tractable metabolic vulnerability [222]. Clinically oriented cohort studies and risk models also connect ferroptosis-associated gene patterns with prognosis and drug resistance phenotypes in ALL. In T-ALL, pharmacological perturbation can drive cells toward ferroptosis, for instance, dihydroartemisinin promotes this process by suppressing SLC7A11-mediated antioxidant defense and activating ER-stress signaling [223], while LCN2 depletion enhances sensitivity to GPX4 inhibition (RSL3) [224], reinforcing the importance of iron-handling nodes in setting ferroptosis thresholds.

Across lymphoid malignancies, integrative dependency mapping supports the concept that B-cell tumors can be unusually constrained by anti-ferroptotic defenses, motivating ferroptosis-inducing or -sensitizing strategies in difficult-to-treat disease [225]. In diffuse large B-cell lymphoma (DLBCL) specifically, epigenetic control of ferroptosis resistance is underscored by evidence that BRD4 sustains FSP1 expression; BET inhibition therefore sensitizes GCB-DLBCL to ferroptosis-inducing agents [226]. Additional regulators act at the lipid remodeling layer: TCP1 can modulate ferroptosis sensitivity by stabilizing ACSL4, linking chaperone activity to PUFA-phospholipid programming and patient prognosis [227]. Finally, translational interest is strengthened by prognostic modeling associating ferroptosis gene signatures with immune infiltration patterns and therapeutic resistance states in DLBCL cohorts [228].

### Other solid tumors

In breast cancer, susceptibility to ferroptosis is strongly shaped by cellular plasticity and membrane lipid composition. A seminal study showed that therapy-resistant, high-mesenchymal cancer cell states (including EMT-like programs seen in breast cancer models) become disproportionately dependent on a lipid-peroxide detoxification pathway converging on GPX4, creating a selective susceptibility to GPX4 inhibition [6]. In triple-negative breast cancer (TNBC), multi-omics profiling further revealed substantial ferroptosis heterogeneity across TNBC subtypes and proposed ferroptosis-informed combination strategies with immunotherapy [185]. Consistently, mechanistic and translational studies indicate that disrupting the SLC7A11-GSH-GPX4 axis can preferentially eliminate more therapy-resistant TNBC populations and modulate treatment responsiveness [229].

In prostate cancer, sex-hormone signaling directly intersects with ferroptosis surveillance by regulating phospholipid remodeling enzymes. A recent study identified MBOAT1/2 as ferroptosis suppressors whose protective function can be independent of GPX4 and is differentially controlled by estrogen and androgen receptor signaling [94], implicating hormone-driven membrane remodeling as a key determinant of ferroptosis sensitivity in hormone-responsive tumors.

In hepatocellular carcinoma (HCC), ferroptosis is closely connected to clinical therapy and drug resistance mechanisms. Sorafenib has been reported to induce ferroptosis in HCC, while tumor-intrinsic pathways that preserve lipid homeostasis and antioxidant buffering can blunt this effect; for example, the HBXIP/SCD axis was shown to restrain sorafenib-triggered ferroptosis by promoting fatty-acid desaturation/lipid remodeling, thereby reducing sorafenib efficacy [230]. Broader syntheses of the field emphasize that sorafenib-associated ferroptosis and resistance are frequently shaped by System Xc⁻/SLC7A11-GSH-GPX4 control and interconnected stress-response pathways [231]. In other solid tumors such as ovarian cancer, renal cell carcinoma, and melanoma, converging evidence indicates that lineage-specific lipid/iron metabolism and antioxidant networks create exploitable ferroptosis vulnerabilities [232, 233], supporting the use of tailored ferroptosis‑inducing or sensitizing regimens in selected molecular contexts.

Collectively, studies across diverse cancer types indicate that ferroptosis sensitivity is not uniformly distributed but instead reflects lineage-specific metabolic and redox programs. Tumors characterized by high iron dependency, enriched polyunsaturated phospholipid content, and mesenchymal or therapy-resistant states such as pancreatic ductal adenocarcinoma, triple-negative breast cancer, clear cell renal carcinoma, and certain subtypes of lung cancer tend to exhibit heightened vulnerability to ferroptosis induction. In contrast, cancers with constitutively active antioxidant defenses, including NRF2-driven glutathione synthesis, lipid remodeling toward monounsaturated fatty acids, or robust GPX4/FSP1/DHODH-dependent protection, frequently display intrinsic or acquired ferroptosis resistance.

Importantly, ferroptosis susceptibility is further shaped by tumor-specific contexts, including oncogenic mutations (e.g., KRAS, TP53, KEAP1), tissue oxygenation status, immune infiltration, and metabolic interactions within the tumor microenvironment. These findings suggest that ferroptosis represents a context-dependent vulnerability rather than a universal liability, emphasizing the necessity of cancer-type and state-specific stratification when considering ferroptosis-based therapeutic strategies. Future efforts integrating molecular profiling, ferroptosis biomarkers, and functional metabolic states will be essential for translating ferroptosis targeting into precision oncology.

## Ferroptosis in tumor microenvironment

### Immune cells regulate ferroptosis

Much of the recent research on ferroptosis has focused on cell lines or xenograft models, leaving the role of the tumor microenvironment (TME) comparatively underexplored. Comprising a very complex network, the TME is composed of cancer cells, immune cells (T cells, B cells, macrophages, natural killer (NK) cells, dendritic cells (DCs), myeloid-derived suppressor cells (MDSCs)), stromal cells, fibroblast, adipocytes, endothelial cells, and the extracellular matrix, which together influence cancer progression and therapeutic responses. Fortunately, recent studies have increased the understanding of the relationship between the TME and ferroptosis [234]; notably, interferon gamma (IFNγ) released from effector CD8(+) T cells has been demonstrated to induce lipid peroxidation and ferroptosis by decreasing the expression of SLC7A11 and SLC3A2 and limiting cystine uptake by cancer cells [10].

Recent work from the Zou laboratory demonstrated that TME-derived IFN-γ (from CTLs) and arachidonic acid (AA) synergistically promote tumor cell ferroptosis by activating ACSL4-dependent lipid remodeling [235], implying that targeting ACSL4 could sensitize tumors to immune checkpoint therapy. Furthermore, neutrophils in the TME induced GBM cell ferroptosis by transferring myeloperoxidase-containing granules to tumor cells; however, human GBM studies indicated that ferroptosis plays a protumorigenic role and predicts poor prognoses [236]. Ferroptosis leads to the release of damage associated with molecular patterns (DAMPs) or lipid peroxides, which is considered a proinflammatory process. Extracellular components mediate TME-ferroptosis crosstalk. For example, lactate, a key TME metabolite, regulates lipid biosynthesis and oxidative stress resistance [237]. Mechanistically, extracellular lactate is not only the main carbon source for lipid metabolism but is also a regulator lipid formation mediated through the AMPK pathway [238]. In addition, in liver cancer cells, lactate uptake promoted ATP production to upregulate sterol regulatory element-binding protein 1 (SREBP1) and stearoyl-coenzyme A (CoA) desaturase-1 (SCD1) expression, leading to an increase in MUFA biosynthesis and alleviation of lipid peroxidation and ferroptosis [239]. Therefore, erastin- and RSL3-induced ferroptosis of tumor cells can be inhibited by elevated levels of extracellular lactate [239]. In contrast, the exposure of ferroptotic tumor cells to calreticulin accelerated the maturation of DCs and the infiltration of CTLs into tumors [240]. In the proinflammatory TME, nitric oxide promotes an M1 like macrophage phenotype and confers ferroptosis resistance by partially inhibiting 15-LOX activity [241]. HMGB1 released from ferroptotic cancer cells was an important factor that triggered inflammation and in turn affected immune surveillance. Moreover, an established TME characteristic, hypoxia has recently been shown to protect malignant mesothelioma cells from ferroptosis through the upregulation of carbonic anhydrase 9 (CAS9) expression [242]. Similarly, hypoxia protected primary human macrophages and fibrosarcoma HT1080 cells from ferroptosis through the inhibition of ferritinophagy mediated by NCOA4-dependent and NCOA4-independent mechanisms [243]. However, HIF pathways have been demonstrated to be positive factors for inducing ferroptosis in CCC [58].

### Immune cells promote tumor cell evasion

Importantly, ferroptosis suppression induced by regulating the levels of pivotal molecules in the TME may promote tumor cell escape immune surveillance. In castration-resistant prostate cancer, heterogeneous nuclear ribonucleoprotein L (HNRNPL) promotes immune evasion by both inhibiting T cell-induced tumor ferroptosis and activating the YY1/PD-L1 axis [244]. Interestingly, fatty acids in the TME can induce CD8(+) T-cell ferroptosis in a CD36-dependent manner, suggesting that the downregulation of CD36 expression and the inhibition of CD8(+) T-cell ferroptosis may benefit CD8(+) T-cell-based cancer immunotherapy [89]. In addition, high TYRO3 expression in melanoma and 4T1 mammary carcinoma cells resulted in resistance to anti-PD1/PD-L1 therapy and inhibited tumor cell ferroptosis by facilitating the development of a tumorigenesis microenvironment and tumor cell immune evasion [245]. In addition, DCs induced ferroptosis through PPARG-mediated lipid metabolism, inhibiting the antitumor immune response in mice and promoting a microenvironment conducive to tumor cell immune evasion [98]. In regulatory T cells (Tregs), GPX4 prevents lipid peroxidation and ferroptosis, thereby maintaining Treg suppressive function and blunting antitumor immunity [246]. This recent finding demonstrates that the deletion of *Gpx4* in Tregs is a potential therapeutic strategy to potentiate cancer immunotherapy [246]. Moreover, ferroptotic tumor cells release factors that suppress antitumor immunity and promote immune evasion. For instance, the release of 8-hydroxy-2′-deoxyguanosine (8-OHdG) by ferroptotic tumor cells resulted in infiltration by tumor-associated macrophages (TAMs) triggered via the activation of the STING-dependent DNA sensor pathway and M2 macrophage polarization, thus promoting pancreatic tumorigenesis [221]. Prostaglandin E2 (PGE2), a key immunosuppressive factor released by ferroptotic cancer cells, suppressed the immune functions of NK cells, cytotoxic T cells and DCs [124, 247]. Additionally, other lipid peroxides released by ferroptotic cancer cells before or after disintegration, including hydroperoxyeicosatetraenoic acid (15-HpETE-PE), 5-HpETE, 4-HNE and their derivatives, may negatively affect antitumor immune responses by inhibiting DC maturation and antigen presentation or by inducing immune cell ferroptosis [234]. However, few studies on the function of these aforementioned lipid products derived from ferroptotic cells have been reported, indicating the need for further extensive studies on these compounds. Collectively, these findings link ferroptosis to tumor immune evasion, highlighting the importance of finely regulating ferroptosis in the development of TME-targeted immunotherapies.

### Exosomes modulate ferroptosis-related antitumor immunity

Exosomes are critical communication vehicles in the TME, shuttling proteins, lipids, and non-coding RNAs between cancer cells, immune cells, and stem cells to influence tumor progression and therapy [248]. Notably, the GC cell-derived exosomal long noncoding (lnc) RNA ENDOG-1:1 (lncFERO) controlled the stemness of gastric cancer stem cells (GCSCs) by directly interacting with SCD1 mRNA and recruiting heterogeneous nuclear ribonucleoprotein A1 (hnRNPA1), which contributed to the inhibition of ferroptosis and acquired chemoresistance of GC cells [249]. Similarly, cells have been shown to resist ferroptotic stress by inducing the expression of Prominin2, which promotes the formation of exosomes that transport iron in ferritin out of cells [29]. In contrast, macrophage-derived extracellular vesicles contributed to mesothelial carcinogenesis induced by asbestos through ferritin intake [250]. Another report indicated that the oncoprotein KRAS^G12D^ released from ferroptotic cancer cell-derived exosomes was absorbed by macrophages, leading to polarization of the macrophages through STAT3, which promoted tumor growth [251]. In addition, cancer-associated fibroblast (CAF)-secreted exosomal miR-522 inhibited the ferroptosis of GC cells and promoted acquired chemoresistance [252]. Based on these leading findings, engineered exosome-like nanovesicles have been developed to trigger high and specific CTL immune responses against multiple types of cancer cell by inducing tumor cells ferroptosis and reprogramming the TME [92]. Moreover, several nanocarrier strategies involving exosomes have been developed to activate the antitumor immune response and increase the ferroptosis rate of multiple types of cancer cells [253]. Although current evidence linking exosomes to ferroptosis is limited and their roles in the TME are complex, elucidating how exosomes modulate ferroptosis-related antitumor immunity remains a promising research direction.

**Fig. 7:**
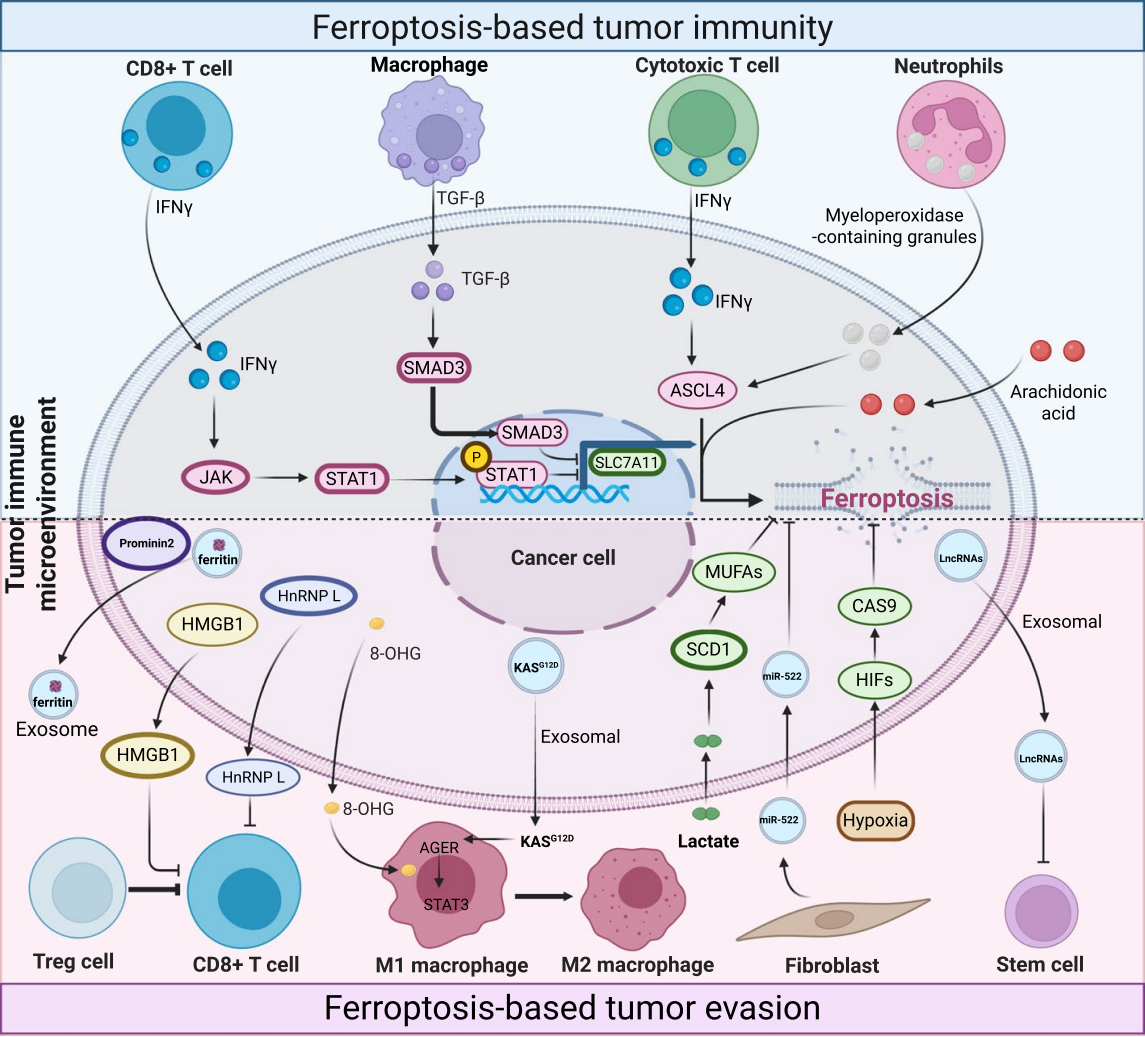
Role played by ferroptosis in the tumor microenvironment and antitumor immunity. Ferroptosis plays a complex role in the TME by influencing antitumor immunity, antitumor response efficacy and tumor cell evasion. On the one hand, immune cells can release inflammatory cytokines, which promote tumor cell ferroptosis. For example, IFN-γ released by CD8(+) T cells triggers tumor cell ferroptosis by activating the JAK-STAT1 signaling pathway, which suppresses the expression of SLC7A11. Immune cell-induced tumor cell ferroptosis can therefore be leveraged to enhance the potential of antitumor immunotherapy. On the other hand, immune cells undergo ferroptosis, which dampens their antitumor immune response, resulting in tumor cell evasion. Therefore, inhibited ferroptosis of immune cells may help to restore immunological function, including normal immune surveillance, to prevent tumor cell escape. In addition, extracellular substances and conditions in the TME affect the sensitivity of tumor cells to ferroptosis induction. For instance, lactate, nitric oxide and hypoxia protect tumor cells from ferroptosis through distinct mechanisms. Moreover, exosomes secreted by tumor cells, immune cells or tumor-related fibroblasts are important signaling regulators involved in ferroptosis and the TME, which merits further investigation. Overall, the induction or inhibition of tumor cell ferroptosis needs to be determined on the basis of cancer type due to TME plasticity

Recent evidence indicates that immune cells in the TME share metabolic features with cancer cells, rendering them similarly vulnerable to ferroptosis induction [254]. The characteristics related the TME and ferroptosis make exploration into tumor cell susceptibility without affecting antitumor immunity a challenge. The complicated crosstalk between ferroptosis and TME components, as well as with immunotherapy-related factors, has been recently summarized and discussed in an opinion article [234]. Thus, our understanding of how the TME modulates ferroptosis and its therapeutic implications remains incomplete (Fig. 7). Extensive studies are further required to illuminate the regulatory mechanism of cancer immunity induced by ferroptotic therapies, which may promote the discovery of promising combined therapeutic approaches to overcome challenges to TME-related antitumor effects.

## Therapeutic approaches targeting ferroptosis

**Fig. 8:**
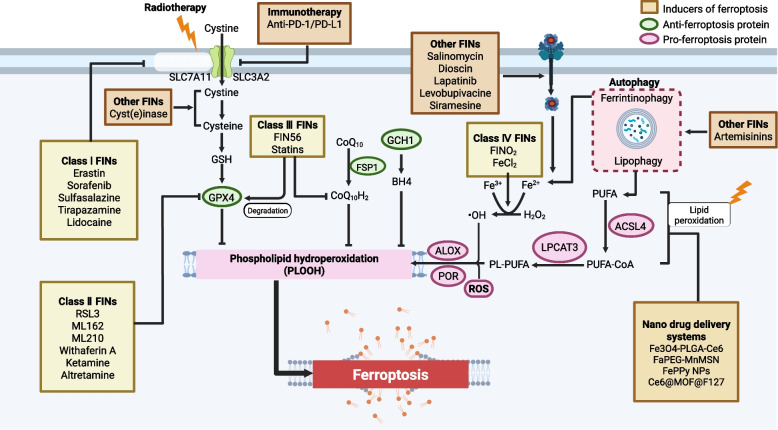
Overview of ferroptosis inducers (FINs) and treatments in cancer therapy. FINs currently used in tumor therapy include small molecules, natural products, nanomaterials and other treatments, such as radiotherapy and immunotherapy. Chemical and natural molecules are categorized into four classes, from I to Ⅳ, based on the targeted ferroptosis regulators. Class I FINs inhibition system Xc^—^ action, class Ⅱ FINs inhibit GPX4 action, class Ⅲ FINs accelerate GPX4 degradation and inhibit ubiquinone activity, and class Ⅳ FINs oxidize iron. In addition, radiotherapy induces the ferroptosis of certain cancer cells via various mechanisms, such as the generation of excessive reactive oxygen species (ROS), inhibition of the Xc^—^ system, and depletion of GSH. Immunotherapies such as anti-PD-1/PD-L1 therapy can also trigger ferroptosis in indicated cancer cells by inducing IFN-γ release by CD8(+) T cells, which suppresses SLC7A11 activity and induces ferroptosis. Moreover, numerous nanomaterials have been designed and synthesized to boost the efficacy of known FINs or induce ferroptosis through multiple targeted mechanisms, including antioxidant defenses, lipid ROS and iron overload

Ferroptosis has been linked to cancer therapy from the very beginning of this developing field, which initiated the discovery of FINs, which were identified through the search for new anticancer drugs [255]. Growing evidence demonstrates that inducing ferroptosis with various compounds in preclinical studies represents a potential therapeutic strategy against certain therapy-resistant tumors. Furthermore, many studies have confirmed that ferroptosis is at least partially involved in several conventional cancer therapies, such as chemotherapy, radiotherapy, immunotherapy and targeted therapy [256]. Specifically, FINs may enhance the efficacy of these conventional therapies by promoting ferroptosis in tumor cells. Additionally, in recent years, many nanomaterials have been successfully developed to overcome difficulties in cancer therapies through the enhancement of FIN activity or the precise delivery of FINs [256]. Herein, we discuss the promising ferroptosis-based approaches for promoting the translation of FIN-related strategies to eliminate cancers (Fig. 8 and Table 1).

**Table 1:** Ferroptosis inducers (FINs) and treatments in cancer therapy

FINs/Approach	Target/Mechanism	Tumor type	Preclinical/Clinical development	Outcomes	Reference
**Ferroptosis inducers (FINs)-Class Ⅰ**					
Erastin	System Xc −	Multiple cancers	NA: not available	NA: not available	[3]
Sorafenib	System Xc −	AML, HCC, neuroblastoma, NSCLC, RCC	Marketed	Synergistically improve anticancer effect with the use of FINs	[285–287]
Sulfasalazine	SLC7A11	Lymphomas, pancreatic cancer, and lung cancer, breast cancer, glioblastoma, head and neck cancer	Marketed as an anti-inflammatory agent, in phase I for the treatment of cancer	Ferroptosis induction in vitro and in mouse tumor-xenograft model	[288]
Imidazole ketone erastin (IKE)	System Xc −	Diffuse large B cell lymphoma (DLBCL)	In vitro and in vivo tests	IKE inhibited tumor growth inDLBCL mouse model via ferroptosis	[258]
Tirapazamine	System Xc −	Osteosarcoma (143B cells)	In vitro study	NA: not available	[266]
Lidocaine	System Xc −, iron accumulation	Ovarian and breast cancer	In vitro and in vivo tests	Ferroptosis induction in lidocaine-treated ovarian and breast cancer cells in vitro and in vivo	[289]
**Ferroptosis inducers (FINs)-Class Ⅱ**					
Altretamine	GPX4	Lymphoma, sarcoma	Marketed	NA: not available	[264]
Withaferin A	GPX4 (KEAP1 inactivation), HO-1	Breast cancer, neuroblastoma, osteosarcoma, hepatocellular carcinoma cells	Phase II	Only grade 1/2 AEs, but no grade ≥ 3 AEs observed	[276, 290]
1S,3R-RSL3	GPX4	head and neck cancer (HNC), non-small cell lung cancer, treatment-resistant prostate cancer cells	Preclinical tests	Ferroptosis induction in vitro and suppressed multiple tumors growth	[46, 291, 292]
DPI7 (ML162); DPI10 (ML210)	GPX4, Nrf2-ARE pathway	Head and neck cancer (HN3R, HN3-rslR)	In vitro studies	NA: not available	[293, 294]
Ketamine	KAT5-GPX4 axis; lncPVT1/miR-214-3p/GPX4 axis	Breast cancer (MCF-7, T47D), liver cancer (HepG2, Huh7)	Preclinical tests	Ketamine significantly suppressed cancer cells in vitro and in vivo by stimulating ferroptosis	[269, 295]
**Ferroptosis inducers (FINs)-Class Ⅲ**					
FIN56	GPX4, ubiquinone, clockophagy	Bladder cancer cells, glioblastoma, fibrosarcoma	Preclinical tests	Ferroptosis induction in vitro and in multiple tumor mice models	[174, 178, 296]
Statins	HMG-CoA reductase (HMGCR), CoQ10	Breast cancer (MDA-MB-231), AML (acute myeloid leukaemia), hepatocellular carcinoma (HCC4006), MM (multiple myeloma)	In vitro studies; Marketed as lipid-lowering agent, in oncology phase I trials	NA: not available	[6, 70, 297]
**Ferroptosis inducers (FINs)-Class Ⅳ**					
FINO_2_	Ferrous iron and lipidome; Inactivation of GPX4	Hepatoma (HT-1080)	In vitro study	NA: not available	[265]
Ferrous ammonium sulfate	Iron; inhibition of GPX4-GSS/GSR-GGT axis	Neuroblastoma (IMR-32)	In vitro tests	NA: not available	[276]
Ammonium ferric citrate	Iron	Non-small-cell lung carcinoma (A549, HCC827)	In vitro evaluation	NA: not available	[298]
Neratinib	Iron	Breast cancer (SKBR3, MCF-7)	Marketed	Neratinib inhibited tumor growth and metastasis, and prolonged survival when used as a neoadjuvant therapy	[299]
Salinomycin	Iron; Iron sequestration in lysosome	Various solid tumor types	In vitro and in vivo tests; Marketed as anticoccidial drug	NA: not available	[300, 301]
Lapatinib + siramesine	Iron; Increase intracellular iron level causing oxidative damage	Breast cancer (MDA MB 231, MCF-7, ZR-75 and SKBr3); Glioma (U87); Lung adenocarcinoma (A549)	In vitro tests	Ferroptosis occurred in breast cancer, glioma and lung adenocarcinoma cells	[302, 303]
**Ferroptosis inducers (FINs)-Others**					
DPI2	GSH, GPX4	Hepatoma (BJeLR cells)	In vitro tests	Ferroptosis induction	[124]
Buthionine sulfoximine	GSH (glutamate-cysteine ligase)	Melanoma; Ovarian cancer	Phase I	Grade 1/2 nausea and/or vomiting in 50% of patients; Appropriate biochemical dose: 13 g/m^2^	[304]
Temozolomide	Accumulation of lipid peroxides; DMT1	Glioblastoma (Glioblastoma stem cells (GSCs), TG905)	Preclinical tests	Ferroptosis was detected in GSCs	[205, 305]
Curcumin and its analogue	Heme oxygenase-1 (HO-1/HMOX1); Accumulation of intracellular iron via autophagy; Lipid peroxidation	Breast cancer cells, non-small-cell lung cancer, osteosarcoma cells	In vitro tests	Ferroptosis induction in targeted cancer cells	[306–308]
Artemisinins	Glutathione S-transferase; Altering cellular iron homeostasis	Lung cancer; PDAC cell lines, head and neck carcinoma cells, leukemia cells	Preclinical tests	Ferroptosis was detected	[309, 310]
Cyst(e)inase	Cysteine depletion	Prostate cancer (DU145 and PC3), breast cancer (MDA-MB-361), human chronic lymphocytic leukemia	In vitro and in vivo tests	Cyst(e)inase suppressed the growth of multiple tumors and prolonged median survival time of mice	[311]
BAY 87–2243	Inhibition of mitochondrial complex I; Combined necroptotic/ferroptotic cell death	Melanoma (A375, BAY 87–2243, G-361 cells)	In vitro and in vivo tests	BAY 87–2243 significantly reduced tumor growth in various BRAF mutant melanoma mouse xenografts and patient-derived melanoma mouse models	[312]
Apatinib	SREBP-1a and GPX4	Gastric cancer (MGC-803 cells, multi-drug-resistant GC cells)	In vitro and in vivo mouse tumor xenograft model evaluations	Ferroptosis induction and significant reductions in tumor volumes and tumor weights	[313]
Baicalin	FTH1	Bladder cancer (5637 and KU-19–19 cells)	In vitro and in vivo tests	Ferroptosis induction in bladder cancer cells and xenograft model	[289]
Cefotaxime	HMOX1	Nasopharyngeal carcinoma (CNE2 cells)	In vitro and in vivo tests	Cephalosporin antibiotics exert specific and selective anticancer effects on nasopharyngeal carcinoma mainly via HMOX1 overexpression-induced ferroptosis	[269]
Dihydroisotanshinone I	GPX4-dependent ferroptosis, apoptosis	Lung cancer (A549 cells and H460 cells)	In vitro and in vivo tests	Lung cancer cells growth inhibition via ferroptosis and metastasis suppression in mice	[314]
Dioscin	Lipid ROS and intracellular irons	Melanoma cells (A375, WM115)	In vitro tests	Ferroptosis induction in discocin-treated melanoma cells	[268]
Talaroconvolutin A	SLC7A11, ALOXE3	Colorectal cancer cells (HCT116, SW480, SW620)	In vitro and in vivo tests	Talaroconvolutin A suppressed the growth of xenografted colorectal cancer cells via ferroptosis	[315]
Elesclomol	ATP7A, SLC7A11degradation	Colorectal cancer (SW480, DLD-1)	In vitro and in vivo tests	Elesclomol inhibited colorectal cancer both in vitro and in vivo	[316]
Erianin	Calcium/calmodulin signaling	Lung cancer (H460, H1299)	In vitro and in vivo tests	Erianin inhibits lung cancer cell growth and migration via ferroptosis	[317]
Glycyrrhetinic acid	NADPH oxidases and iNOS	Triple-negative breast cancer cells	In vitro study	Glycyrrhetinic acid triggered ferroptosis in triple-negative breast cancer cells	[318]
α-eleostearic acid	Acyl-CoA synthetase long-chain isoform 1 (ACSL1)	Multiple cancer cells and triple-negative breast cancer cells	In vitro and in vivo tests	α-eleostearic acid limited TNBC xenograft growth and metastasis with markers of ferroptosis	[49]
Flubendazole	P53, lipid peroxidation	Castration-resistant prostate cancer (PC3 cells)	In vitro and in vivo tests	Flubendazole promotes the ferroptosis in CRPC tumor xenografts	[319]
Gambogenic acid	p53/SLC7A11/GPX4 signaling pathway	Melanoma (A375 and A2058 cells)	In vitro study	NA: not available	[320]
DMOCPTL(derivative of natural product parthenolide)	Ubiquitination of GPX4	Triple negative breast cancer cells	In vitro and in vivo tests	DMOCPTL induced ferroptosis in TNBC cells, inhibited the growth of breast tumor and prolonged the lifespan of mice, with no observed side effects	[321]
Jiyuan oridonin A	GPX4	Gastric cancer (MGC-803)	In vitro and in Patient-derived tumor xenograft models tests	Jiyuan oridonin A inhibited the growth of gastric cancer cells and suppressed patient-derived tumor in xenograft models	[322]
Cucurbitacin B	GPX4, lipid peroxidation	Nasopharyngeal cancer (CNE1)	In vitro and in vivo tests	Cucurbitacin B inhibited tumor progression without causing obvious side effects	[323]
Juglone	Iron accumulation, heme degradation	Endometrial carcinoma (Ishikawa cells)	In vitro test	Ferroptosis detected in Juglone-treated Ishikawa cells	[324]
Metformin	Iron accumulation, lipid ROS, SLC7A11 degradation	Breast cancer (MCF7 and T47D cells)	In vitro and in vivo tests	Ferroptosis induction in vitro and in vivo	[325]
Nitroprusside	NA: not available	Melanoma (B16)	In vitro test	NA: not available	[326]
Phyto-sesquiterpene lactone, DET, and its derivative, DETD-35	GPX4 inhibition	Melanoma (A375, A375-R)	In vitro test	Ferroptosis induction in vitro	[327]
Pseudolaric acid B	GSH depletion, p53-mediated xCT pathway	Glioma (C6, SHG-44, U251, U87)	In vitro and in vivo tests	Ferroptosis induction	[328]
Punicic Acid	Lipid peroxidation	Carcinoma (HCT-116) and hypopharyngeal carcinoma (FaDu)	In vitro and in vivo tests	Ferroptosis detected in carcinoma cells and in vivo	[329]
Quercetin	p53-independent lipid peroxidation, ferritin degradation	A variety of cancer cells	In vitro study	NA: not available	[330]
Formosanin C	Ferritinophagy	Human hepatocellular carcinoma (HepG2 and Hep3B cells)	In vitro study	NA: not available	[331]
Shuganning injection	HO-1, labile iron pool accumulation	Triple-negative breast cancer (MDA-MB-231 and MDA-MB- 468)	In vitro and in vivo tests	Ferroptosis induction in vitro and in vivo	[332]
Solasonine	GPX4	Hepatoma carcinoma (HepG2 and HepRG cells)	In vitro and in vivo tests	Solasonine suppressed tumor growth via ferroptosis induction	[333]
Tagitinin C	PERK-Nrf2-HO-1 signaling pathway	Colorectal cancer (HCT116 cells)	In vitro study	NA: not available	[334]
**Immunotherapy**					
Anti-PD-1/PD-L1	HnRNP L inhibition and CD8 + T cell infiltration	Prostate cancer	Preclinical tests	HnRNP L knockdown enhanced antitumor immunity by recruiting infiltrating CD8^+^ T cells and synergized with anti-PD-1 therapy in CRPC tumors	[244]
BEBT-908	p53, antitumor immune responses	Human diffuse large B-cell lymphoma (Daudi), non-small cell lung cancer (H2122), colorectal cancer (HCT116 and MC38)	Preclinical tests	BEBT-908 that potently inhibits tumor cell growth and potentiates anti-PD1 therapy in mice by inducing immunogenic ferroptosis in cancer cells	[295]
T cell-derived interferon (IFN)γ + arachidonic acid (AA)	ACSL4; Lipid metabolism	Yumm5.2 cells, MC38 cells, A375 cells	Preclinical tests and correlation analysis with clinical melanoma patients received adoptive T cell therapy (ACT)	Clinically, tumor ACSL4 correlates with T cell signatures and improved survival in immune checkpoint blockade (ICB)-treated cancer patients	[235]
**Radiotherapy**					
Ionizing radiation	ACSL4, SLC7A11 and GPX4, p53	HT-1080, H460, A549, and H1299 cell lines	Preclinical tests and the evaluation patient-derived xenograft tumors	Radiotherapy induces ferroptosis in cancer patients, and correlated with better response and longer survival in cancer patients	[9, 142, 272]
**Nano-materials**					
Coordination polymer	Multiple targets (Fenton reaction, GSH depletion, GPX4 inhibition, chemotherapy, starvation therapy)	Various cell lines and in vivo tumor models (LL2, A549, MCF-7, CT26, B16F10, 4T1 and HT-1080)	In vitro and in vivo tests	Ferroptosis induction in vitro and in vivo	[275]
MOFs	Multiple mechanisms (Fenton reaction, GSH depletion, improvement of hypoxia, PTT)	A variety of cancer cells and in vivo tumor models (4T1, Huh-7, MDA-MB-231)	In vitro and in vivo tests	Ferroptosis induction in vitro and in vivo	[335]
Metal nanoparticles	Multiple targets (Fenton reaction, GSH depletion, SLC7A11 inhibition, Amino acid starvation, enhanced lipid peroxidation)	Varied cancer cells and in vivo tumor models (4T1, SAS, MCF-7/ADR,)	In vitro and in vivo tests	Ferroptosis induction in vitro and in vivo	[336]
Self-assembled nanoparticles	Multiple mechanism (Fenton reaction, GSH depletion, SLC7A11 inhibition, increased lipid peroxide, autophagy, PTT, immunotherapy)	Multiple cell lines and in vivo tumor models (HepG2, 4T1, CAL-27)	In vitro and in vivo tests	Ferroptosis induction in vitro and in vivo	[337]
Metallic oxide	Core mechanisms (GSH depletion, GPX4 inhibition, SLC7A11 inhibition, Fenton reaction, autophagy)	A variety of cancer cells and in vivo tumor models (U87MG, 4T1, CT26, HepG2, HCT-116, HeLa, MDA-MB-231)	In vitro and in vivo tests	Ferroptosis induction in vitro and in vivo	[275]
Liposome	Chemotherapy, Fenton reaction, SLC7A11 inhibition GSH depletion	Varied cancer cells and in vivo tumor models (4T1, 3T3, Huh7, MDAMB-231)	In vitro and in vivo tests	Ferroptosis induction in vitro and in vivo	[338]

### Direct inducers of ferroptosis

Since ferroptosis was first defined in the cancer field, numerous chemicals and treatments have been discovered to trigger the ferroptosis of malignant cells. Based on the mechanisms outlined above, highly potent small-molecule and natural compounds that induce ferroptosis have been classified into at least four categories. Class Ⅰ FINs target SLC7A11 activity and inhibit the uptake of cystine and the generation of cysteine, which is required for glutathione synthesis [257]. The most commonly used class Ⅰ FIN is erastin; however, erastin has been mostly used for in vitro studies due to its low water solubility and metabolic stability. Subsequently, an analog of erastin named imidazole ketone erastin was designed with enhanced ferroptosis inducing potency in vivo and has shown superior solubility and stability to suppress tumor growth [258]. Another class Ⅰ FIN is sulfasalazine, an FDA-approved anti-inflammatory drug, that promotes tumor cell death by targeting SLC7A11 activity. Although sulfasalazine shows relatively poor metabolic stability and potency, its ferroptosis-promoting activity has been verified in patients with malignant glioma [259], and in combination with radiotherapy. Sulfasalazine is being evaluated in a clinical trial as a glioblastoma treatment (NCT04205357). Recently, a phase I clinical trial was conducted to evaluate sulfasalazine efficacy and safety in breast cancer patients (NCT03847311). Similarly, another drug, SRF, approved for the treatment of multiple types of cancers via the inhibition of various kinases has recently been demonstrated to induce ferroptosis partially through the suppression of SLC7A11 activity [260]; however, the classification of SRF as a class FIN is debatable due to its nonferroptotic cellular effects [261]. In addition to these small molecules, an engineered human enzyme, cyst(e)inase, has been demonstrated to induce ferroptosis by degrading cysteine and cystine (cyst(e)ine) in pancreatic tumors [115], EGFR-mutant NSCLC [137], and breast cancer [17]. Intriguingly, cyst(e)inase exhibited antitumor activities by promoting ferroptosis when used in combination with ROS-inducing drugs or immune checkpoint inhibitors [10]. Targeting cyst(e)ine degradation via cyst(e)inase may represent a novel strategy and offer a new avenue for developing cancer therapies based on ferroptosis induction. Furthermore, class Ⅱ FINs exert antitumor effects by directly or indirectly blocking GPX4 activity, and best characterized FINs are RSL3, ML162 and ML210. RSL3 and ML162 both include a highly reactive chloroacetamide group and covalently bind to the selenocysteine residue not only in GPX4 but also of most other selenoproteins [262], which have limited their in vivo applications [263]. ML210 is a nitroisoxazole-containing compound and is a prodrug from which α-nitroketoxime (named JKE-1674) was generated, and JKE-1674 forms a nitrile-oxide electrophile by which it covalently binds to GPX4 in cancer cells with clearly improved specificity [263]. Because the genetic deletion of GPX4 is lethal to mice, the use of GPX4 inhibitors for cancer therapy remains a challenge due to safety concerns. However, the FDA-approved anticancer agent altretamine and the natural anticancer agent withaferin A have been demonstrated to target GPX4 and decrease GPX4 levels in vivo [264], suggesting an alternative for regulating GPX4 in the clinical therapy of tumors. In addition, class III FINs include FIN56, which accelerates the degradation of GPX4 and inhibits ubiquinone activity, resulting in ferroptosis [70]. Class Ⅳ FINs, such as FINO_2_, directly oxidize ferrous iron and indirectly inactivate GPX4 to induce ferroptosis [265]. Although the pro-ferroptotic effects of class III and IV FINs are evident in cultured cells, their efficacy in vivo awaits evaluation.

In addition to the aforementioned well-characterized FINs, an increasing amount of evidence showed that numerous chemicals induce ferroptosis in a context-dependent manner. For example, temozolomide may have suppressed GBM cell growth partially by inducing ferroptosis via targeted DMT1 expression [205]. Tirapazamine inhibits the proliferation and migration of osteosarcoma cells and induces ferroptosis in part by inhibiting SLC7A11 activity [266]. Cephalosporin antibiotics exert specific and selective anticancer effects on nasopharyngeal cancer (NPC) mainly via HMOX1 overexpression-induced ferroptosis [267]. Dioscin induces melanoma cell ferroptosis by affecting the expression of transferrin and ferroportin, which are regulators of intracellular iron levels [268]. Moreover, some anesthetics induce the ferroptosis of certain cancer cells. Levobupivacaine treatment remarkably elevated the levels of ROS and irons in NSCLC cells. Mechanistically, levobupivacaine upregulated the expression of p53 and induces ferroptosis by regulating p53 expression in NSCLC cells [268]. Guan-Nan [269] determined that ketamine suppressed the viability of liver cancer cells and induced ferroptosis; they identified lncPVT1/miR-214-3p/GPX4 axis involvement in the possible regulatory mechanism.

### Radiotherapy induces ferroptosis

Ferroptosis has recently been demonstrated to play a key role in radiotherapy-induced tumor growth suppression, which challenges the conventional theory that radiotherapy triggers apoptosis and DNA damage in cancer cells [11]. A growing number of studies support the notion that ferroptosis contributes to the anticancer effects of radiotherapy [270]. Herein, we discuss the latest advances in ferroptosis-based radiotherapy for overcoming cancer resistance and explore combination therapeutic strategies to augment investigations into ferroptosis induction in cancer therapy. Excessive ROS generated by radiotherapy can damage intracellular biomolecules such as lipids, which are potentially linked to lipid peroxidation and ferroptosis. Radiotherapy-induced ferroptosis has been extensively investigated and implicated in the death of several types of cancer cells, including lung cancer (H460, H1299 and A549 cell lines), breast cancer, esophageal cancer (KYSE 30 and KYSE 150 cell lines), renal cell carcinoma, ovarian cancer, vulvar cancer, fibrosarcoma (HT1080 cell line), and melanoma cells [9, 270–272]. Mechanistically, radiotherapy triggers excessive lipid peroxidation and ferroptosis through at least three parallel pathways [272]. First, radiotherapy induces excessive ROS, which lead to lipid peroxidation through the transfer of electrons (hydrogen atoms) released by PUFAs, forming PUFA radicals. Second, radiotherapy elevates ASCL4 expression, which accelerates the biosynthesis of PUFA-PLs and the subsequent oxidative damage and ferroptosis of cancer cells [9]. Third, as a core component of the DNA repair system, ATM-mediated SLC7A11 downregulation and subsequent GSH depletion partially contribute to radiotherapy-induced ferroptosis by weakening GPX-dependent antioxidant defenses [11, 272]. In addition, DNA damage activates the STING1/TMEM173-mediated DNA-sensing pathway, which results in the autophagy-dependent ferroptosis of human pancreatic cancer cells [273], suggesting that the activation of other DNA-sensing pathways may play a similar role in promoting radiotherapy-related ferroptosis. Although the exact underlying mechanisms of radiotherapy-induced ferroptosis remain largely unknown, the leverage of ferroptosis in tumor radiotherapy is an important ongoing area of investigation. Thus, accumulating evidence supports a strong link between ferroptosis and tumor radiotherapy, warranting further studies to determine how best to modulate ferroptotic pathways for maximal clinical benefit. Another important factor is the intrinsic or acquired radioresistance of tumors, which has been a long-standing challenge in the radiotherapy field; however, the induction of ferroptosis has recently been linked to radiosensitization, providing an alternative for combination therapies that eliminate resistant cancer cells. For example, radiotherapy generally upregulates the expression of SLC7A11 and GPX4 in an adaptive response that protects cancer cells from ferroptotic induction, rendering cells radiosensitive. Therefore, the downregulation or deletion of SLC7A11/GPX4 can significantly enhance radiosensitization by amplifying radiation-induced ferroptosis [9]. Several preclinical studies have indicated that FINs synergize with radiotherapy via the perturbation of antiferroptotic regulators during cancer treatment [9, 11, 272], suggesting that administration of FINs targeting ferroptotic regulatory molecules to promote radiotherapy-induced ferroptosis may be a promising strategy for radiosensitization in the clinic. In summary, recognizing the critical role of ferroptosis in tumor radiotherapy, a deeper understanding of its relationship with radioresistance and radiosensitization is needed. This may reveal opportunities to develop therapeutic strategies that target ferroptosis during radiotherapy.

### Immunotherapy and ferroptosis

As discussed above, ferroptosis-based immunotherapy through the application of immune checkpoint inhibitors (ICIs) such as anti-PD-1/PD-L1 leads to potential benefits for eradicating refractory cancers. Anti-PD-L1 therapy increased lipid ROS levels and the ferroptosis rate in ovarian tumors through activation of CD8(+) T cells, and the combination of cyst(e)inase and PD-L1 blockade exerted synergistic effects to promote ferroptosis [10]. In addition, ferroptotic cancer cells enhanced immunogenicity by activating immune responses to tumors, boosting the efficacy of immunotherapies [240]. Similarly, early ferroptotic cancer cells have been shown to promote the phenotypic maturation of DCs and stimulate a vaccination-like effect in vitro and in vivo [274]. These obvious natural advantages of ferroptosis-related immunotherapy have encouraged investigation into the immune regulatory effects of ferroptotic cancer cells and potential combination therapies based on ferroptosis induction. However, ferroptotic tumor cells can suppress antitumor immune responses by releasing immunomodulators, including 8-OHdG, PGE2 and oxidized PLs, enhancing the TME [221, 241]. Moreover, although FINs enhanced the efficacy of ICIs in several preclinical studies [10], a combination therapy strategy may not be suitable under all conditions due to the distinct immunophenotypes in the TME. Indeed, immune cells exhibit varied responses to different FINs, such as GPX4 inhibitors or Xc^−^ system inhibitors [234]. Hence, specific FINs that combine with ICIs should be considered on the basis of the specific immune characteristics. More importantly, FINs can suppress antitumor immune cells such as CD8(+) T cells and NK cells, resulting in defects in antitumor immunity, which can limit the efficacy of ICIs [89]. From this perspective, ferroptosis inhibitors could represent a promising strategy to enhance antitumor immunity. However, this idea is based on preliminary evidence showing that ferrostatin-1 protects CD8(+) T cells from ferroptosis and improves ICI efficacy [89]. Furthermore, the TME establishes ferroptosis-promoting condition due to abundant fatty acids, high redox stress and other factors, resulting in the vulnerability of immune cells to ferroptosis [89]. The application of ferroptotic inhibitors might be helpful for the functional recovery of immune cells in the TME to maintain antitumor immunity. Further studies are needed to define the contexts and optimal timing for using ferroptosis inhibitors to reactivate antitumor immunity by blocking ferroptosis in immune cells. Taken together, the available data demonstrate the dual role played by ferroptosis in antitumor immunotherapy and the complicated crosstalk between immune cells and tumor cells in the TME. Therefore, to achieve the maximal potential of ferroptosis-inducing strategies in tumor immunotherapy, several issues remain to be elucidated through future investigations. First, the regulatory mechanism of increased tumor immunity mediated by ferroptotic inducers must be known; specifically, the distinct responses of immune cells to different FINs need to be elucidated. Second, the cancer modalities most sensitive to ferroptosis when treated with ICIs and FINs need to be identified in clinical settings. Third, further studies are necessary to establish the FIN and temporal administration to use for enhancing the immunogenicity of tumor cells or to eliminate immunosuppressive cells in the TME. The answers to these questions will provide the insights necessary for the development of immunotherapy or combined antitumor therapies based on ferroptosis induction for certain cancers.

### Ferroptosis and nanotechnology

Inducing ferroptosis has attracted considerable interest as a potential strategy to eradicate treatment-resistant cancers using various FINs. However, the clinical efficacy of FIN monotherapy remains limited, largely due to tumor complexity and heterogeneity. To address these limitations, ferroptosis-driven nanomedicine has emerged in recent years as a promising alternative approach [275]. An increasing number of studies have demonstrated the potential of ferroptosis-based nanobiotechnology and combined tumor therapy. Herein, we summarize the latest advances in this promising field that combines ferroptosis and antitumor nanotechnology. First, the best FINs exhibit low potency, solubility and pharmacokinetic parameters, which have limited their translation to clinical trials [125]. Nanomaterials that function as drug carriers may aid in improving the efficacy of established FINs and developing novel inducers of ferroptosis for antitumor therapy. For instance, the erastin analog IKE showed enhanced antileukemia effects when it was delivered by polyethylene glycol-poly(lactic-co-glycolic acid) nanoparticles [258]. In addition, the poor solubility and low safety profile of the FIN withaferin A was improved by utilizing amphiphilic degradable pH-sensitive nanocarriers, which specifically enhanced the efficacy of withaferin A in high-risk neuroblastoma [276]. Similarly, a growing number of nanomaterials have been developed as nanocarriers for delivering FINs as antitumor therapeutics, and they showed enhanced physiochemical properties, drug availability, targeted delivery, pharmacokinetics and efficacy and reduced off-target toxicity [277]. Second, emerging nanotherapeutics have been designed mostly based on an increase in the intracellular iron level and lipid peroxidation rate in malignant cells, which has been achieved mainly through the following strategies: (1) targeting iron metabolism to boost the Fenton reaction and subsequent lipid peroxidation, (2) disrupting the antioxidant defense system and limiting the production of ROS scavengers, and (3) delivering lipids into tumor cells and rewriting lipid metabolism in the TME [275, 278]. For example, iron oxide nanoparticles, iron-platinum nanoparticles, polymeric micelles incorporating iron, amorphous nanometallic glass and metal organic frameworks (MOFs) have been extensively investigated as tools to suppress various tumors by promoting excessive iron accumulation and inducing subsequent ferroptosis. In addition to the direct delivery of iron into tumor cells, the regulation of iron metabolism-related proteins or receptors by nanomaterials has contributed to ferroptosis induction. For example, a Tf-based nanodrug delivery system not only triggered ferroptosis by releasing Fe^3+^ but also localized to tumor cells through the Tf receptor-mediated endocytosis pathway [279]. Recently, nanomaterials targeting the Xc^—^ system, inhibiting GPX4 activity and depleting GSH have been widely investigated to efficiently induce ferroptosis for cancer treatment. Moreover, to generate excessive lipid peroxides, AA and unsaturated lipid-rich phosphatidylcholine have been used as nanoparticles, such as liposomes and copolymers, to encapsulate FINs and promote lipid peroxidation and esterification of PUFAs, increasing the cancer cells ferroptosis rate [280]. In addition to direct delivery of lipids into tumor cells, heat stress induced by a polypeptide-modified and 1H-perfluoropentane (1H-PFP)-encapsulated Fe_3_O_4_-containing nanoformulation (GBP@Fe_3_O_4_) reprogrammed lipid metabolism and promoted cancer cell ferroptosis [281]. Third, the complicated TME characterized by acidity, hypoxia, heterogeneity and oxidative stress limits the efficacy of currently available FINs in vivo [234], suggesting the need to design multifunctional nanomaterials to overcome these difficulties in antitumor therapies. Therefore, various TME-responsive nanomaterials have been ingeniously developed to enhance the therapeutic effects of FINs when used in combination with other therapies, including photothermal therapy (PTT), photodynamic therapy (PDT), chemodynamic therapy and imaging-guided therapeutics [278]. For example, a nanoplatform consisting of mesoporous carbon NPs (MCNs), triiron dodecacarbonyl (FeCO) and doxorubicin (DOX) showed multifunctional effects against breast tumors by releasing CO under the guidance of photoacoustic imaging and releasing DOX to induce ferroptosis at tumor site in an acidic environment, exhibiting enhanced therapeutic efficacy in vivo [282]. Moreover, a multifunctional nanosystem based on a ferroptosis induction strategy combined with PTT has been prepared using a nitrogen-coordinated carbon-supported Pd SAzyme, and it clearly responded to a unique TME to generate a mild increase in TME temperature, subsequently inducing ∙OH accumulation, GPX4 inactivation and, ultimately, ferroptosis [283]. Similarly, ferroptosis induced in combination with PDT has emerged as a potential strategy to overcome difficulties in cancer therapy. A novel hypoxia-responsive nanoreactor BCFe@SRF with SRF, constructed by covalently connecting chlorin e6-conjugated bovine serum albumin (BSA-Ce6) and ferritin through an azobenzene (Azo) linker, was prepared and showed unmatched potential for highly efficient synergistic PDT and ferroptosis effects [284]. Mechanistically, BCFe@SRF is degraded in a hypoxic environment, which releases BSA-Ce6 to trigger PDT upon laser beam exposure, ferritin to induce the iron-catalyzed Fenton reaction, and SRF to induce tumor antioxidative defense disruption [284]. In summary, significant progress has been made in ferroptosis-based nanotherapeutics for cancer in recent years. However, their clinical translation remains challenging, primarily due to safety concerns. Further studies are required to evaluate the long-term effects of nanodrug delivery systems on human health and to translate nanotherapeutics based on ferroptosis to clinical oncology settings.

### Combination therapies and ferroptosis

The induction of ferroptosis has emerged as a promising strategy to enhance the efficacy of conventional cancer therapies. A growing body of evidence indicates that ferroptosis acts as a critical cell death modality triggered by various standard treatments, including chemotherapy, radiotherapy, immunotherapy, and targeted therapies. Consequently, combining ferroptosis inducers (FINs) with these conventional approaches can synergistically augment tumor cell death, overcome therapy resistance, and improve treatment outcomes.

Several chemotherapeutic agents and radiotherapy (RT) have been shown to induce ferroptosis, in part through the accumulation of lipid peroxides. For instance, cisplatin and doxorubicin can promote ferroptotic death in cancer cells, and this effect is potentiated by co-treatment with FINs such as erastin or sulfasalazine, which inhibit the cystine/glutamate antiporter system Xc⁻ (SLC7A11) [339]. Similarly, ionizing radiation promotes lipid peroxidation and ferroptosis, an effect that is often counteracted by adaptive upregulation of SLC7A11 or GPX4 in cancer cells [11]. In preclinical models of non-small cell lung cancer (NSCLC) and other malignancies, combining RT with FINs that target SLC7A11 or GPX4 can radiosensitize tumors and enhance ferroptosis [9]. Notably, in tumors with intrinsic radioresistance such as those harboring TP53 or KEAP1 mutations, adding FINs can restore radiosensitivity by inducing ferroptosis [142].

The intersection between ferroptosis and antitumor immunity has opened new avenues for combination strategies. CD8(+) T cell-derived interferon-γ (IFNγ) downregulates SLC7A11 expression in tumor cells, thereby promoting ferroptosis [10]. This mechanism provides a rationale for combining immune checkpoint inhibitors (ICIs) with FINs. Preclinical studies indicate that inhibiting SLC7A11 synergizes with anti-PD-1/PD-L1 therapy to enhance T cell-mediated tumor killing and ferroptosis [340]. Moreover, ferroptotic cells release immunostimulatory damage-associated molecular patterns (DAMPs) such as HMGB1 and ATP, which can promote dendritic cell maturation and amplify antitumor immune responses [341]. Interestingly, some immunosuppressive cells in the tumor microenvironment, including M2-like tumor-associated macrophages (TAMs) and regulatory T cells (Tregs), show differential susceptibility to ferroptosis, offering a window to selectively eliminate these cells while sparing antitumor immune effectors [197].

Resistance to molecularly targeted agents often involves the adaptive activation of pathways that defend against ferroptosis. For example, in EGFR-mutant NSCLC, cancer cells become highly dependent on cystine uptake, and SLC7A11 inhibition synergizes with EGFR tyrosine kinase inhibitors to induce ferroptosis [342]. Similarly, in hepatocellular carcinoma treated with sorafenib, upregulation of metallothionein-1G (MT1G) confers ferroptosis resistance; combining sorafenib with MT1G inhibition restores ferroptosis and overcomes drug resistance [343]. In KRAS-driven tumors, which often upregulate SLC7A11 and glutathione synthesis, FINs targeting system Xc⁻ show potent efficacy alone or in combination with MEK inhibitors [344]. Despite promising preclinical data, the clinical translation of ferroptosis-based combinations requires careful consideration of therapeutic windows and normal tissue toxicity. Several clinical trials are currently evaluating FINs (e.g., sulfasalazine, cyst(e)inase) in combination with chemotherapy, RT, or immunotherapy in cancers including glioblastoma, NSCLC, and pancreatic cancer (NCT04205357, NCT04092647) [345, 346]. In summary, combining FINs with conventional therapies constitutes a multifaceted strategy to target ferroptosis as a key vulnerability in cancer. By leveraging the unique metabolic vulnerabilities of tumor cells and the immunomodulatory effects of ferroptosis, these combinations hold significant potential to improve therapeutic responses and overcome resistance. Future efforts should focus on optimizing drug delivery, validating biomarkers, and elucidating context-dependent mechanisms to maximize efficacy and safety in clinical settings.

### Re-sensitizing resistant cancers to ferroptosis

Cancer cells exploit a variety of adaptive responses to evade ferroptosis, including upregulating antioxidant defenses, rewiring lipid metabolism, and activating pro-survival signaling pathways. Recent research has identified several promising strategies to overcome such resistance and re-sensitize tumors to ferroptosis induction, offering new avenues for combination therapies.

Several oncoproteins and signaling cascades promote ferroptosis evasion. For instance, the ubiquitin ligase NEDD4 is upregulated in melanoma and limits erastin-induced ferroptosis by downregulating VDAC2/3 expression; its inhibition restores sensitivity to system Xc⁻ blockade [347]. Beyond the roles of specific E3 ligases, the proteasome stress-response program can itself regulate sensitivity to ferroptosis. Recent studies show that activation of the DNA-damage inducible 1 homolog 2 (DDI2) and nuclear factor erythroid-2 derived,-like-1 (NFE2L1) axis upregulates the ubiquitin–proteasome system, thereby protecting cells from ferroptosis [348]. This identifies enhanced proteostasis as a potential resistance mechanism and a co-targetable node for re-sensitizing therapy-refractory tumors to FINs. Similarly, DJ1 (PARK7), often overexpressed in cancers, protects cells from ferroptosis by maintaining the activity of S-adenosyl homocysteine hydrolase (SAHH) in the transsulfuration pathway, which supports cysteine synthesis independent of SLC7A11. Genetic or pharmacological inhibition of DJ1 sensitizes tumors to ferroptosis inducers (FINs) [349]. Pyruvate dehydrogenase kinase 4 (PDK4), which is upregulated in pancreatic ductal adenocarcinoma, suppresses ferroptosis by shifting metabolism toward fatty acid synthesis; PDK4 inhibition re-sensitizes tumors to SLC7A11 inhibitors [75]. These examples highlight that directly inhibiting key resistance-driving proteins can restore ferroptosis sensitivity.

Many resistant cancers upregulate core components of ferroptosis defense, such as SLC7A11, GPX4, FSP1, or GCH1. Targeting these systems especially in tumors that become dependent on them can effectively re-establish vulnerability. For example, in KRAS-mutant cancers, which often exhibit heightened SLC7A11 expression and glutathione synthesis, cysteine-depleting agents (e.g., cyst(e)inase) or SLC7A11 inhibitors potently induce ferroptosis and suppress tumor growth [115]. In tumors with activated PI3K-AKT-mTORC1 signaling, a common driver of ferroptosis resistance, combining mTORC1 inhibitors with FINs synergistically triggers ferroptosis and impairs tumor progression [350]. Additionally, cancers with low basal expression of GPX4-independent defense components are highly dependent on GPX4; in such contexts, GPX4 inhibition alone can effectively induce ferroptosis [127]. Conversely, tumors with low GPX4 expression are vulnerable to inhibition of FSP1 or DHODH, revealing a synthetic-lethal interaction between parallel ferroptosis defense arms [127, 129].

Dysregulation of lipid composition and iron metabolism is central to ferroptosis resistance. Some cancers downregulate peroxidizable polyunsaturated fatty acid-containing phospholipids (PUFA-PLs) or enhance the degradation of peroxidized lipids to avoid ferroptosis. Calcium-independent phospholipase A₂β (iPLA₂β), overexpressed in p53-wild-type cancers, hydrolyzes peroxidized PLs and promotes resistance; iPLA₂β inhibition sensitizes cells to ferroptotic stimuli [351]. The adipokine chemerin, upregulated in renal cell carcinoma, reduces peroxidized PUFA-PL levels and protects tumors from ferroptosis; neutralizing chemerin enhances ferroptosis sensitivity [352]. Iron metabolism is also frequently dysregulated: overexpression of iron–sulfur cluster assembly proteins such as NFS1 limits the labile iron pool and desensitizes cancer cells to ferroptosis. Inhibiting these proteins increases intracellular free iron and resensitizes tumors to FINs [17]. Similarly, prominin 2-mediated iron export via ferritin-containing vesicles protects matrix-detached breast cancer cells from ferroptosis; blocking this pathway restores sensitivity [29].

Re-sensitizing resistant cancers to ferroptosis requires a nuanced understanding of the specific adaptive mechanisms in tumor cells. Strategies include co-targeting upstream oncogenic signals, inhibiting overactive ferroptosis defense pathways, modulating lipid and iron metabolism, and exploiting metabolic rewiring linked to resistant phenotypes. Promising preclinical data support the combination of FINs with targeted agents, metabolic inhibitors, or conventional therapies to overcome resistance. Moving forward, the identification of predictive biomarkers such as expression levels of SLC7A11, FSP1, GPX4, or key lipid-metabolizing enzymes will be essential for selecting patients most likely to benefit from these approaches. With several candidates already in clinical testing, ferroptosis-sensitizing strategies hold significant potential to expand the therapeutic arsenal against recalcitrant and resistant cancers.

## Clinical research progress targeting ferroptosis

Translating ferroptosis induction from a compelling preclinical concept into a viable clinical strategy is a frontier in oncology. The intrinsic vulnerability of certain cancer types, particularly those with mesenchymal or therapy-resistant phenotypes, to ferroptosis, coupled with the detailed mapping of its core machinery, has spurred clinical investigation into pharmacological agents that trigger this iron-dependent cell death. These agents, often repurposed from other therapeutic areas or developed as novel compounds, target key nodes within the ferroptosis pathway, offering new avenues to combat refractory cancers [256, 353]. This section outlines the current clinical progress of ferroptosis-inducing drugs for cancer treatment, highlighting their mechanisms, trial stages, and therapeutic contexts.

### Clinical trials of ferroptosis inducers

The clinical exploration of ferroptosis inducers (FINs) leverages multiple strategies to disrupt cellular redox balance. A prominent approach involves inhibiting the system Xc − cystine/glutamate antiporter, leading to glutathione (GSH) depletion and consequent inactivation of the master regulator glutathione peroxidase 4 (GPX4). Sorafenib, a multi-kinase inhibitor standardly used for hepatocellular carcinoma (HCC) and renal cell carcinoma, is a prime example. In addition to its kinase targets, sorafenib potently inhibits system Xc −, inducing ferroptosis which contributes to its therapeutic efficacy [231]. Resistance often emerges via upregulation of SLC7A11, the catalytic subunit of system Xc −, highlighting the centrality of this axis. Similarly, sulfasalazine, an anti-inflammatory drug, acts as a system Xc − inhibitor and has entered early-phase oncology trials for glioblastoma and other solid tumors, showing potential to re-sensitize tumors to conventional therapies [354, 355]. The engineered enzyme cyst(e)inase, which degrades extracellular cysteine and cystine to deplete cellular GSH, has demonstrated potent ferroptosis induction and tumor suppression in preclinical pancreatic cancer models, paving its way toward clinical translation [115].

Directly targeting GPX4 is another key strategy. While specific GPX4 inhibitors such as RSL3 remain in preclinical development, several chemotherapeutic agents exert part of their cytotoxicity by inactivating GPX4. Cisplatin, a cornerstone of chemotherapy for various solid tumors, induces ferroptosis by depleting GSH and inactivating GPX4, providing a crucial mechanism to overcome apoptotic resistance [339]. The nucleoside analog gemcitabine, widely used in pancreatic and other cancers, has been linked to ferroptosis induction via the HSPA5/GPX4 pathway, and its efficacy can be enhanced by co-treatment with other FINs [126]. Furthermore, natural compounds like withaferin A are under investigation for their ability to target and inactivate GPX4, showing promise in high-risk neuroblastoma and HCC models [276, 290].

Modulating iron metabolism and lipid peroxidation directly offers additional clinical entry points. Artesunate and its derivative dihydroartemisinin, well-known antimalarials, promote ferritinophagy to increase intracellular labile iron, driving Fenton chemistry and lipid peroxidation. They are being evaluated in phase II trials for various cancers and show synergistic effects with sorafenib in HCC [356, 357]. Lapatinib and neratinib, tyrosine kinase inhibitors for HER2-positive breast cancer, have been found to elevate intracellular iron and promote ferroptosis, which may underlie their activity against brain metastases and in overcoming resistance [299, 358].

Notably, several commonly used drug classes have been repurposed as FINs. Statins (e.g., simvastatin, lovastatin), inhibitors of HMG-CoA reductase in the mevalonate pathway, deplete CoQ10 and promote GPX4 degradation, inducing ferroptosis [297]. Their excellent safety profile makes them attractive candidates for combination therapies, with trials exploring their role in modulating the tumor immune microenvironment [359]. The antipsychotic haloperidol, a dopamine receptor D2 (DRD2) antagonist, induces autophagy-mediated ferroptosis and synergizes with temozolomide in glioblastoma models, warranting its investigation in oncology [360].

The most promising clinical applications likely lie in combination therapies. Ferroptosis induction synergizes powerfully with immunotherapy. Activated CD8(+) T cells release IFNγ, which downregulates SLC7A11 and upregulates ACSL4 in tumor cells, thereby sensitizing them to ferroptosis [10]. Combining checkpoint inhibitors (e.g., anti-PD-1) with FINs (e.g., GPX4 inhibitors, sulfasalazine) has shown enhanced antitumor efficacy in preclinical immunocompetent models by fostering an immunogenic tumor microenvironment [185]. Similarly, radiotherapy induces ferroptosis through DNA damage-mediated SLC7A11 suppression and ACSL4 upregulation [11]. Combining FINs with radiotherapy synergistically increases lipid peroxidation and overcomes radioresistance, a strategy under active preclinical and translational investigation [9]. Despite the enthusiam, clinical translation faces challenges, including the context-dependent roles of ferroptosis (which may promote tumorigenesis early on), its potential to impair antitumor immunity in some settings, and the lack of highly specific, bioavailable FINs with optimal pharmacokinetics. Future trials will need to carefully select patient populations based on biomarkers of ferroptosis sensitivity (e.g., high ACSL4, low GPX4 or FSP1 expression), determine optimal therapeutic sequences and combinations, and monitor both on-target efficacy and potential immune-related consequences. The table (Table 2) below summarizes key drugs with ferroptosis-inducing activity that have entered clinical cancer trials, illustrating the diversity of agents and targets being explored.

**Table 2:** Selected clinical trial drugs inducing ferroptosis for cancer treatment

Drugs	Cancer type	Target/Mechanism	Clinical trial number	Clinical trial status	References
Sorafenib	AML, HCC, neuroblastoma, NSCLC, RCC	System Xc −	NCT03794440 NCT03247088 NCT02559778 NCT00064350	Marketed	[24, 272, 361, 362]
Sulfasalazine	Lymphomas, pancreatic cancer, and lung cancer, breast cancer, glioblastoma, head and neck cancer	SLC7A11	NCT04205357	Marketed as an anti-inflammatory agent; phase I in cancer	[9, 354, 363–365]
Altretamine	Lymphoma, sarcoma	GPX4	NCT00002936	FDA-approved drug, but in vivo concentrations may not be sufficient to inhibit GPX4	[264]
Zalcitabine	Pancreatic cancer, AIDS-related Kaposi sarcoma	DNA stress	NCT00000954	Marketed for the treatment of HIV, phase I for the treatment of cancer	[273]
Withaferin A	Recurrent Ovarian Cancer, breast cancer, neuroblastoma, osteosarcoma,	GPX4 HO-1	NCT05610735	Phase II	[276, 290]
Simvastatin	Multiple myeloma	HMGCR	NCT00281476	phase II trials	[297]
Neratinib	Breast cancer	Iron	NCT03377387 NCT03457896	Marketed	[358]
Lovastatin	Prostate cancer, ovarian cancer	HMGCR	NCT00585052	Marketed as lipid-lowering agents, in oncology	[359]
Buthionine sulfoximine	Melanoma, neuroblastoma,ovarian cancer	GSH	NCT00005835 NCT00002730	Phase I	[304, 366, 367]
Artesunate	non‑Hodgkin lymphoma, hepatocellular carcinoma	Iron	NCT00764036 NCT03093129	Marketed as an antimalarial drug, in oncology phase II trials	[356, 368]
Lapatinib	Breast cancer	Iron	NCT03085368 NCT00356811 NCT00667251	Marketed	[302]
Cisplatin	Bladder cancer Cervical cancer Pancreas cancer	GSH	NCT01656551 NCT04574960 NCT01561586 NCT03649321	Marketed	[365, 369–371]
Gemcitabine	Pancreatic cancer, biliary tract cancer, Solid Tumor	GPX4	NCT06015659 NCT05357196 NCT05147272	Marketed	[126, 372]
β-Elemene	Non-small-cell lung cancer, glioblastoma	TFEB	NCT03123484 NCT02629757	Marketed	[373]
Brequinar	Cervical cancer Colon cancer Fibrosarcoma Lung cancer AML	DHODH, Dihydroorotate dehydrogenase	NCT03760666	Phase II	[127, 374, 375]
Carbon Nanoparticle-Loaded Iron	Advanced solid tumor	Iron metabolism	NCT06048367	Phase I	[376]

In conclusion, clinical research targeting ferroptosis is rapidly evolving from random discovery to rational design. By repurposing existing drugs and developing novel agents that cripple the GPX4-centered defense network or amplify iron-dependent lipid peroxidation, investigators are opening new therapeutic windows against aggressive and therapy-resistant cancers. The integration of ferroptosis inducers with established modalities like immunotherapy and radiotherapy represents a particularly promising paradigm, poised to improve patient outcomes in the near future.

### Biomarkers for ferroptosis and patient stratification

The clinical translation of ferroptosis as an oncology therapeutic strategy hinges on reliably identifying tumors vulnerable to this cell death modality and stratifying patients most likely to benefit. Ferroptosis biomarkers are thus indispensable tools for guiding treatment decisions, predicting therapeutic response, monitoring efficacy, and understanding resistance mechanisms, reflecting the complex metabolic and redox landscape of ferroptosis. Integrating these biomarkers into clinical practice is critical for advancing precision oncology approaches focused on modulating ferroptosis.

The defining biochemical event of ferroptosis is the iron-dependent peroxidation of polyunsaturated fatty acid-containing phospholipids (PUFA-PLs). Consequently, direct detection of lipid peroxidation (LPO) products serves as a primary biomarker. Sensitive methods include flow cytometry using the fluorescent probe BODIPY 581/591 C11 and immunohistochemical staining for stable end-products like 4-hydroxynonenal (4-HNE) and malondialdehyde (MDA). Recent advancements in lipidomics enable the precise quantification of oxidized phospholipid species, offering a more specific signature of ferroptotic damage [105]. Beyond lipids, the relocalization of transferrin receptor 1 (TFR1) to the plasma membrane is a promising histological marker reflecting increased cellular iron import [4]. The expression levels of key pathway genes provide a genetic basis for stratification. Tumors with high SLC7A11 and GPX4 expression often exhibit robust anti-ferroptotic defenses, while elevated ACSL4 and TFR1 are associated with enhanced sensitivity. A ferroptosis vulnerability signature incorporating the ratio of pro- to anti-ferroptotic gene expression has shown prognostic value [185]. Genomic context is also crucial. For example, tumors harboring KEAP1 mutations or exhibiting NRF2 hyperactivation upregulate a broad antioxidant program, conferring resistance to certain FINs [377].

Proteomic approaches have identified specific protein modifications. A significant discovery is hyperoxidized peroxiredoxin 3 (PRDX3), identified as a specific in vivo marker of ongoing ferroptosis [378]. Metabolic biomarkers are also crucial. Depletion of glutathione (GSH), an increased cysteine/cystine ratio, and elevated levels of labile iron within tumors could identify cancers primed for ferroptosis. Furthermore, circulating lipid peroxidation-derived metabolites or damage-associated molecular patterns (DAMPs) like HMGB1 released from ferroptotic cells are being explored as non-invasive, dynamic biomarkers of treatment response [379].

For monitoring therapeutic response, serial measurement of LPO products or ferroptosis-associated circulating tumor DNA (ctDNA) profiles could track tumor cell death. The upregulation of PTGS2 (COX-2) mRNA is a consistent transcriptional response serving as a useful pharmacodynamic biomarker [380]. Resistance biomarkers are equally important. The induction of FSP1 or GCH1 expression following GPX4 inhibition represents an adaptive resistance mechanism, the detection of which in post-treatment biopsies could guide combination therapy.

The clinical translation of ferroptosis-based therapy will likely require integrated, multimodal biomarker panels that capture genetic, proteomic, metabolic, and lipid peroxidation features to define a practical “ferroptosis susceptibility index”. Key challenges include tumor heterogeneity and the need to standardize assays and thresholds across platforms and centers. Moving forward, the rigorous validation and integration of a robust biomarker arsenal into clinical trials are essential for identifying appropriate patients, optimizing treatment regimens, overcoming resistance, and realizing the full therapeutic potential of targeting ferroptosis in cancer.

## Future perspectives and outstanding questions

As a novel form of RCD, ferroptosis has been extensively explored in recent years. Numerous reports describing mechanistic discoveries and preclinical models have elucidated its molecular and metabolic regulation and underscored its critical role in human diseases, particularly in cancer suppression [4]. Leveraging ferroptosis may be an effective intervention strategy to prevent and treat cancer because cancer cells depend on oncogenic and survival signaling that renders them more susceptible to ferroptosis. As documented herein, factors such as iron accumulation, fatty acid synthesis and the EMT are conducive to tumor progression and increase the vulnerability of various cancer cell types to ferroptosis. Notably, multiple drugs implicated in ferroptosis regulation are already approved for clinical use or under clinical trials. In this context, FDA-approved anticancer agents (altretamine and sorafenib) and FDA-approved non-anticancer drugs (sulfasalazine and statins) may provide opportunities for mechanism-guided repurposing and combination regimens[381]. Emerging evidence is showing that radiotherapy and immunotherapy applied to cancer induce and/or otherwise affect cell response to ferroptosis, suggesting that a combination therapeutic strategies based on ferroptosis induction can be exploited further for the eradication of malignant diseases [270]. Despite significant advances in understanding ferroptosis in cancer therapy over the past five years, translating this knowledge into the clinic requires overcoming specific challenges. Here, we discuss key outstanding questions that must be addressed to advance ferroptosis into the next stage of cancer therapy.

### What are the molecular bases of ferroptosis?

Although considerable progress has been made in understanding ferroptosis, the molecular events in its final execution phase remain largely unknown, particularly how peroxidized phospholipids (PLs) damage membranes to cause cell death. Does the uncontrolled peroxidation of PUFA-PLs account for the ultimate loss of cell membrane integrity during the final stage of ferroptosis? Findings suggest that two PUFA tails in PLs are particularly effective drivers of ferroptosis [71], indicating that lipid cross-linking may result in limited fluidity of membrane components and ultimately lead to ferroptosis. However, other possibilities that may explain pivotal processes, such as reactive electrophiles generated from the decomposition of oxidized PULA-PLs, which probably react with macromolecules and thus damage cell membranes and cause cell death, cannot be discounted.

Recent biophysical studies using single-bilayer systems, including giant or large unilamellar vesicles and supported lipid bilayers, have begun to clarify how PUFA-PL peroxidation alters membrane structure and mechanics. Iron- or ROS-driven oxidation of PUFA-PLs generates hydroperoxides and truncated oxidized phospholipids that loosen lipid packing, increase area per lipid, lower line tension, and facilitate the nucleation and growth of nanometer-scale pores, eventually culminating in catastrophic bilayer rupture [382]. Quantitative imaging and leakage assays further suggest at least two regimes of permeabilization: one dominated by truncated oxidized lipids that form relatively stable aqueous pores, and another in which extensive oxidation leads to membrane thinning, budding and micellization-like disintegration of the bilayer [383]. Lipid-omics studies in these model systems support these observations by showing enrichment of specific oxidized phospholipid species that correlate with pore formation, and related work indicates that oxidized phospholipids can also form covalent adducts and cross-links with membrane proteins, further disturbing membrane architecture. Together, these data provide a mechanistic framework in which PUFA-PL peroxidation alters the mechanical landscape of the bilayer to favor pore formation and loss of barrier function during ferroptosis.

Thus, the precise mechanisms underlying the final execution of ferroptosis in cancer cells, considering their unique membrane and metabolic properties, remain to be fully elucidated. In this context, several other scientific questions remain to be answered in the future: (1) what type of membrane damage is necessary or sufficient to cause ferroptosis; (2) what are the crosstalk signals between ferroptosis and other RCD modalities in fighting against cancer; (3) how do ferroptosis-lysed cancer cells affect the TME; and (4) how does cancer metabolic reprogramming affect ferroptosis?

### How can ferroptosis be leveraged for cancer therapy?

While inducing ferroptosis is an effective way to suppress cancer growth in preclinical models, how to safely and effectively target this process in cancer patients remains a key clinical challenge. First, among the many ferroptosis inducers (FINs) developed in preclinical studies, which are the most promising candidates for clinical trials? To evaluate the efficacy and safety of ferroptotic drug candidates, choosing the best malignant disease model with a large population of patients is a critical factor in future randomized controlled trials. Second, although ferroptosis induction is a potential therapeutic strategy in numerous cancers [381], which tumor type or patient group is most likely to benefit from ferroptosis-promoting therapy? Because tumor cells feature fast growth due to increased iron demand as well as sustained proliferation signals and gene mutation [164], a definitive tumor type and integrated genetic information of the patients may help distinguish malignant diseases that are most susceptible to ferroptotic therapies in the clinic. Third, more effective and accessible biomarkers for reporting ferroptosis induction in patients or at tumor sites are needed, although anti-TfR1 antibodies have been demonstrated to be effective immunological tools to detect cancer cells undergoing ferroptosis [4]. Using ferroptosis biomarkers in biological systems can guide the establishment of pharmaceutical regimens and prevent adverse effects in cancer patients. Finally, we do not know whether cancer cells are resistant to ferroptosis in patients due to long-term use of ferroptosis-promoting drugs. Given the high relapse rates with targeted therapies and the metabolic plasticity of tumor cells, it is likely that cancers will evolve resistance to ferroptosis-inducing therapies. To counteract cancers that develop resistance, the basic molecules that drive ferroptosis resistance need to be identified. Moreover, treatments to address ferroptosis resistance in these cancer patients need to be considered before testing in clinical trials. Therefore, understanding these critical issues during the discovery of ferroptotic therapies to fight cancer will be of tremendous therapeutic value.

### How can ferroptosis be integrated with conventional therapies?

Emerging evidence demonstrates that radiotherapy and immunotherapy can directly induce ferroptosis in cancer cells [11], suggesting that combination therapies leveraging ferroptosis mechanisms could be highly effective. The combination of radiotherapy and chemotherapy potentially depends on ferroptotic enhancement of antitumor effects. Methods to synergize FINs with irradiation in the treatment of malignant cancers will be an important direction in the development of combined anticancer therapies [270]. In addition, the latest findings revealed that ferroptotic inducers not only trigger cancer cell ferroptosis but also change the TME or potentiate the efficacy of immunotherapy [234]. Furthermore, immunotherapy-activated CD8(+) T cells can induce ferroptosis of cancer cells [10], suggesting that modulating immune cells in the tumor microenvironment (TME) may directly engage ferroptotic signaling to suppress tumor growth. Given that immune checkpoint blockade therapy, such as regulation of PD-1/PD-L1, is a pivotal cancer immunotherapy strategy, the combination of immunogenic ferroptosis and cancer immunotherapy may produce a synergetic effect that ultimately eradicates malignant tumors. Therefore, cancer immunotherapy and ferroptosis induction should be considered when exploring effective combined therapies against intractable cancers. The issues relevant to this potential combination therapy of cancer include the following: (1) what are the molecular bases of immunogenic ferroptosis and cancer immunotherapy; (2) which tumor types or patients are susceptible to this combined cancer therapy, and (3) how can small molecules or drug delivery systems that play dual roles in immunogenic ferroptosis induction and immunoregulation be developed? We predict that over the next several years, the mechanisms through which immunogenic ferroptosis is achieved and modulated in numerous cancers will be illuminated. Furthermore, on the basis of ferroptosis and cancer immunotherapy, the development of combined antitumor therapies will shed new light on the potential application of ferroptosis induction in clinical oncology.

### How to balance benefits and risks of ferroptosis-based therapy?

Currently, ferroptosis-dependent pharmacology is a promising tool to realize cancer prevention and therapy. However, before a ferroptosis-targeted strategy can be leveraged to benefit cancer patient health, some challenges need to be addressed. Among known FINs, the best one for in vivo and/or in clinical use needs to be urgently identified. Additionally, small molecules targeting key ferroptotic genes or enzymes induce excessive lipid peroxidation, resulting in the ferroptosis of tumor cells; however, off-target effects are common in animal and human studies. For example, the multikinase inhibitor sorafenib, which also acts as a ferroptosis inducer, causes dose-limiting hepatotoxicity linked to lipid peroxidation and ferroptotic injury in healthy hepatocytes [287]. Likewise, genetic or dietary impairment of lipid antioxidant defenses such as GPX4 deficiency or chronic vitamin E deprivation leads to progressive neurodegeneration and paralysis in experimental models, closely resembling uncontrolled ferroptosis and underscoring the particular vulnerability of the central nervous system [212]. Furthermore, the physiological effects of prolonged activation of lipid peroxidation are unclear in humans, and therapeutic strategies targeting ferroptosis pathways in cancer need to be tissue-specific. Although certain ferroptotic inducers seem to be likely drug candidates for entry in clinical trials, the therapeutic window and toxicity profiles in humans remain to be determined. Comprehensive pharmacological studies are thus required to assess the timing and extent of ferroptotic drug-induced toxicity and to evaluate whether these toxic events can be alleviated through the optimization of drug dosing and scheduling. In the near future, we hope to see bioavailable ferroptosis drug candidates enter clinical trials for treating malignant diseases by their maximizing ferroptosis-promoting strengths and minimizing potential risks to humans.

## Conclusion

In summary, the past several years has witnessed rapid progress in understanding the role of ferroptosis in cancer treatment. Hence, mechanisms by which ferroptosis affects cancer cells have been identified, and novel chemical tools and therapies against malignant cancer have been developed. The knowledge of ferroptosis acquired to date will benefit the prevention, diagnostics and therapies in cancer. The development of translational anticancer therapies targeting ferroptosis depends on a deeper mechanistic understanding of its regulation, which involves complex metabolic interactions and lethal lipid peroxidation. Collectively, exciting findings on pharmacological regulators of ferroptosis hold the potential to translate ferroptosis-based therapies into clinical practice, ultimately improving outcomes for cancer patients. Ferroptosis-based anticancer research promises to realize a wealth of future advances and will continue to be an active field through which understanding and treatment of malignant disease will be accomplished.

## Data Availability

All data generated or analysed during this study are included in this published article and its supplementary information files.
